# Mendelian Randomization Identifies CpG Methylation Sites With Mediation Effects for Genetic Influences on BMD in Peripheral Blood Monocytes

**DOI:** 10.3389/fgene.2020.00060

**Published:** 2020-02-28

**Authors:** Fangtang Yu, Chuan Qiu, Chao Xu, Qing Tian, Lan-Juan Zhao, Li Wu, Hong-Wen Deng, Hui Shen

**Affiliations:** ^1^ Center for Bioinformatics and Genomics, Department of Global Biostatistics and Data Science, School of Public Health and Tropical Medicine, Tulane University, New Orleans, LA, United States; ^2^ School of Basic Medical Science, Central South University, Changsha, China

**Keywords:** osteoporosis, bone mineral density, causal inference, Mendelian randomization, DNA methylation, epigenome-wide association

## Abstract

Osteoporosis is mainly characterized by low bone mineral density (BMD) and is an increasingly serious public health concern. DNA methylation is a major epigenetic mechanism that may contribute to the variation in BMD and may mediate the effects of genetic and environmental factors of osteoporosis. In this study, we performed an epigenome-wide DNA methylation analysis in peripheral blood monocytes of 118 Caucasian women with extreme BMD values. Further, we developed and implemented a novel analytical framework that integrates Mendelian randomization with genetic fine mapping and colocalization to evaluate the causal relationships between DNA methylation and BMD phenotype. We identified 2,188 differentially methylated CpGs (DMCs) between the low and high BMD groups and distinguished 30 DMCs that may mediate the genetic effects on BMD. The causal relationship was further confirmed by eliminating the possibility of horizontal pleiotropy, linkage effect and reverse causality. The fine-mapping analysis determined 25 causal variants that are most likely to affect the methylation levels at these mediator DMCs. The majority of the causal methylation quantitative loci and DMCs reside within cell type-specific histone mark peaks, enhancers, promoters, promoter flanking regions and CTCF binding sites, supporting the regulatory potentials of these loci. The established causal pathways from genetic variant to BMD phenotype mediated by DNA methylation provide a gene list to aid in designing future functional studies and lead to a better understanding of the genetic and epigenetic mechanisms underlying the variation of BMD.

## Introduction

Osteoporosis is a disease that is clinically characterized by an increased risk for fracture due to reduced bone mineral density (BMD) and deterioration of bone quality (Kanis et al., 1994). Osteoporotic fractures represent a major public health problem. Approximately 2 million osteoporotic fractures occur per year in the United States, incurring 17 billion US dollars in direct costs annually, and the burden is estimated to increase by 50% by 2025 (Brent Richards et al., 2012). BMD has a high heritability (50%-85%) as shown in twin and family studies (Arden et al., 2009). Over the past 10 years, numerous genome-wide association studies (GWAS) have identified over 500 genetic loci associated with BMD and other osteoporosis (OP)-related traits (Brent Richards et al., 2012; Estrada et al., 2012; Kemp et al., 2017; Sabik and Farber, 2017; Al-Barghouthi and Farber, 2019; Morris et al., 2019). Although GWAS have been successful in identifying genetic loci for BMD, translating the genetic association findings into knowledge of the underlying biological mechanisms of bone biology and OP remains challenging, primarily due to the fact that most associations are from noncoding variation, the lack of bone-specific-omics resources, and the difficulties in establishing causality between variants, genes, and traits.

DNA methylation is an epigenetic regulation mechanism that plays a key role in many biological processes and disease susceptibility (Grundberg et al., 2013; Gaunt et al., 2016). DNA methylation also affects the differentiation and activities of bone cells (Marini et al., 2016) and contributes to the pathogenesis of OP (van Meurs et al., 2019). Epigenome-wide association studies (EWAS) have been applied to investigate the association between DNA methylation and BMD. The largest EWAS of BMD in bone specimens was performed in 84 postmenopausal women and identified 63 differentially methylated CpGs (DMCs) between healthy and osteoporotic women at a 10% false discovery rate after limiting the association analyses to CpG sites in the top 100 genes whose bone transcripts were previously associated with BMD (Reppe et al., 2017). Due to the difficulties inherent in acquiring bone samples from human subjects, some recent EWAS used whole blood DNA as a proxy to assess the association. The largest EWAS for BMD in whole blood assessed DNA methylation at over 450,000 CpG sites with 5,515 subjects across five cohorts but failed to identify strong consistent association signal at any of the tested CpG sites (Morris et al., 2017). The finding of no significant associations was further substantiated by another independent EWAS of BMD comparing the whole blood DNA methylation of 32 primary OP patients with 16 controls (Fernandez-Rebollo et al., 2018), suggesting that the DNA methylation profile in whole blood may not accurately reflect the epigenomic status of bone cells. Moreover, although EWAS have extensively investigated the associations of DNA methylation and BMD, they were not able to elucidate the role of DNA methylation along the etiology pathway of the genetic variants to OP.

**Figure 1:**
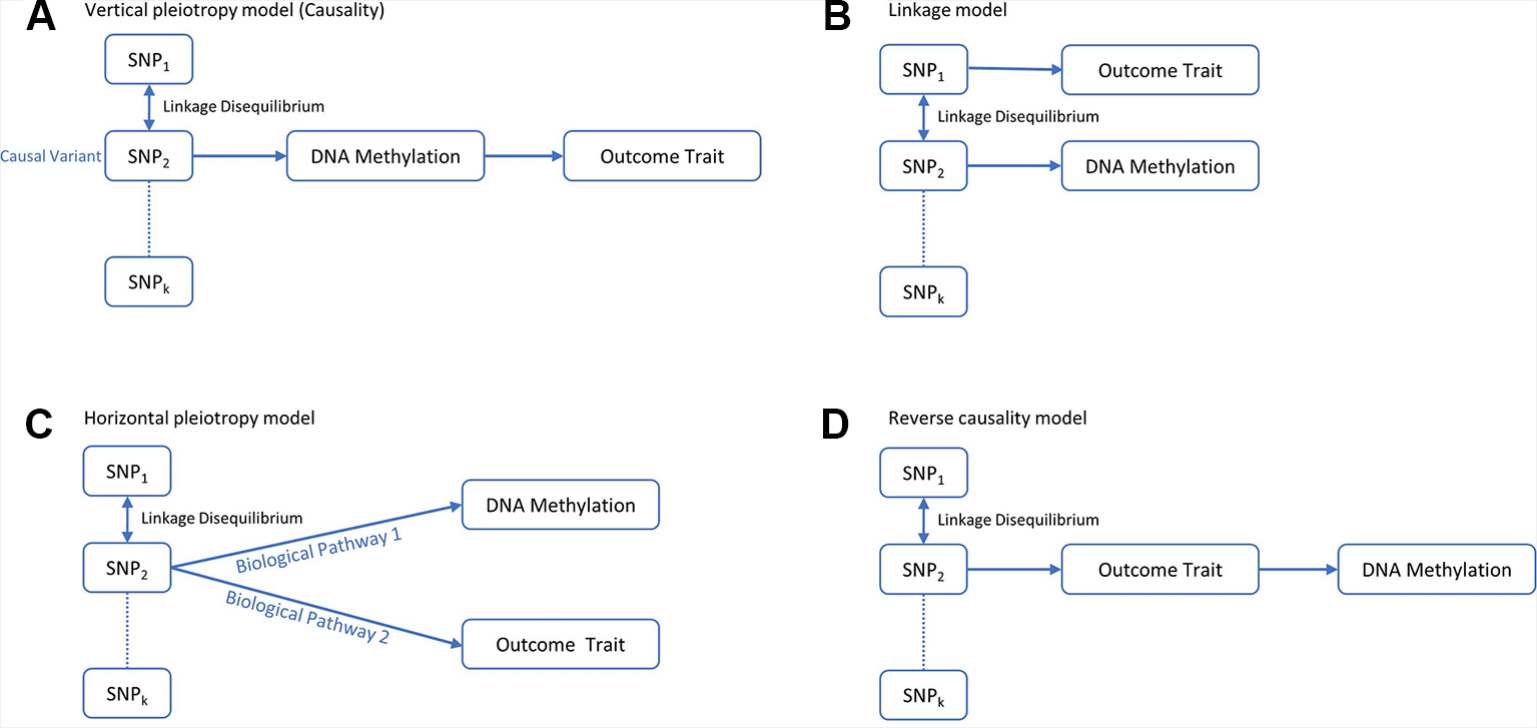
Four possible models for an observed association between a trait and genetic variants through DNA methylation. **(A)** The genetic variant has an effect on the phenotype, mediated through DNA methylation. **(B)** The genetic variant that influences DNA methylation and the variant that influences the associated trait are in LD with each other. **(C)** The genetic variant influences both DNA methylation and phenotype by two independent biological pathways. **(D)** The genetic variant has an effect on the phenotype which then has a downstream effect on DNA methylation at this locus.

**Figure 2:**
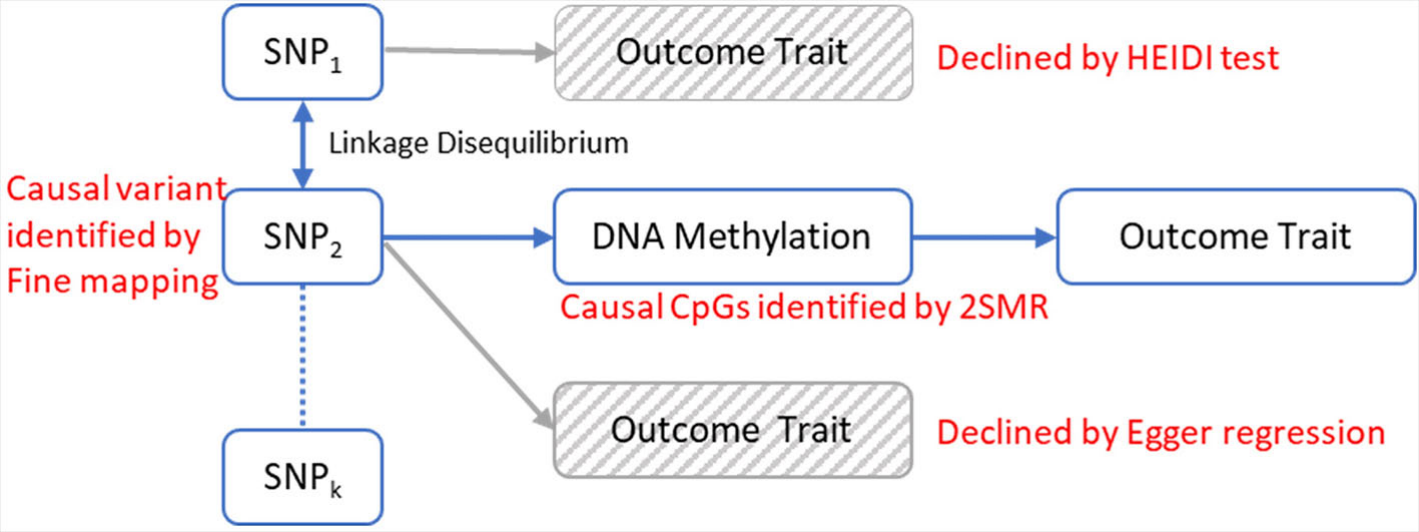
The analytical framework to evaluate the possible models for observed associations between a trait and genetic variants through DNA methylation. The causal relationship between DNA methylation and the trait is established by 2-sample Mendelian randomization (2SMR) with Egger regression to evaluate the horizontal pleiotropy effect. The causal mQTL is identified by fine mapping. The possibility of linkage model is declined by HEIDI test.

Recent studies suggest that DNA methylation modification could reside along the causal pathway between genetic variation and disease, either as the mediating factor contributing to the trait (Richardson et al., 2017) or as a consequence of the trait (Wahl et al., 2017). Identifying the role of epigenetic markers for disease risk along the causal pathway could be valuable in understanding the pathogenic mechanisms of OP. Mendelian randomization (MR) is a statistical method for dealing with this problem. It uses genetic variants robustly associated with modifiable exposures as instrumental variables to infer the causal relationship between the exposure and outcome variable (Davey Smith and Ebrahim, 2003; Davey Smith and Hemani, 2014). Generally, the observed association between a risk factor and an outcome trait can be explained in four kinds of causal pathways: causality, linkage, horizontal pleiotropy and reverse causality (**Figure 1**). Using an MR framework, we can investigate whether DNA methylation resides along the causal pathway to disease (Relton and Davey Smith, 2012). Such an effect is sometimes referred to as “mediation,” as DNA methylation is mediating the effects from genetic variants to phenotype along the same biological pathway. Like other risk factors, DNA methylation is also vulnerable to confounding and reverse causality (Relton and Davey Smith, 2012), but some recently developed methods can help to mitigate these problems. A bidirectional MR framework can distinguish causality from reverse causality (**Figures 1D** and **2**) (Vimaleswaran et al., 2013), a fine-mapping and colocalization framework can identify whether the causal variants are in linkage (**Figures 1D** and **2**), and using multiple correlated instrumental variables (IVs) can distinguish causality from horizontal pleiotropy (**Figures 1C** and **2**) (Burgess et al., 2016; Zhu et al., 2016).

In this study, we built a comprehensive MR framework to evaluate the mediation effect of DNA methylation for genetic influences on traits and applied our framework to discover the genetic causal pathways of OP (**Figure 2**). In our analysis, we first identified the DMCs and methylation quantitative loci (mQTLs) in peripheral blood monocytes (PBMs) from 118 Caucasian women with divergent BMD values and then implemented the MR framework to infer the causal pathways mediated by DMCs. PBMs can serve as circulating precursors of osteoclasts, the bone-resorbing cells (Xing et al., 2005), and produce various cytokines that have profound effects on osteoclast differentiation, activation, and apoptosis (Zhou et al., 2015). Due to the close biological relevance of PBM and bone metabolism, it has been widely utilized as a working cell model for studying the pathogenesis of OP (Liu et al., 2005; Nose et al., 2009; Cao et al., 2014; Deng et al., 2014; Kim et al., 2014) and other skeletal disorders (Nagy et al., 2008; Kwok et al., 2012). We successfully identified 30 CpG sites in PBMs that may reside along the causal pathway from gene to OP and further ascertained 25 genetic loci that have potential causal effects on the change of DNA methylation at those CpG sites by fine mapping.

## Materials and Methods

### Human Subjects and Phenotype

A total of 118 Caucasian women between 20 and 40 years of age were recruited from the subjects participating in our Louisiana Osteoporosis Study, a cross-sectional study with ongoing recruitment to build a large sample pool (~20,000 subjects) and database for research studies of OP and other musculoskeletal diseases/traits (He et al., 2016; Du et al., 2017). The 118 subjects included 64 with relatively high BMD and 54 with relatively low BMD, corresponding to hip BMD Z scores ≥0.8 and ≤−0.8, respectively. Hip BMD was determined as the combined BMD of the femoral neck, trochanter, and intertrochanteric region measured by Hologic Discovery-A DXA (dual energy X-ray absorptiometry) machines (model number: 010-0575, Hologic Inc., Bedford, MA, USA) (Kim et al., 2002). The DXA machine was calibrated daily, and the coefficient of variation (CV) value of the DXA measurements at total hip on Hologic Discovery-A was 1.0%. The BMD Z score was defined as the number of standard deviations a subject's BMD differed from the mean BMD of their age-, gender-, and ethnicity-matched population. The Z-score was reported directly by the Hologic DXA machine which has incorporated the National Health and Nutrition Examination Survey whole body bone data as reference in the system (Kelly et al., 2009; Fan et al., 2014). For each study subject, weight and height were measured using standard procedures, and lifestyle factors (e.g. exercise, alcohol consumption, smoking, etc.) and medical history were assessed by questionnaires. A set of stringent exclusion criteria (Deng et al., 2002) were adopted to minimize potential confounding effects of nongenetic influence (by physiological and pharmacological conditions) on BMD variation and alteration in PBM DNA methylation profile. The detailed characteristics of the study subjects are shown in **Table 1**.

**Table 1:** Baseline characteristics of subjects from the low and high BMD group.

	Low BMD group (s.d.)	High BMD group (s.d.)	*p*-value
**N**	54	64	
**Age**	31.8 (4.80)	31.5 (4.97)	0.76
**Total Hip BMD *Z*-score** **(standardized)**	0.77 (0.06)	1.11 (0.08)	2.2 × 10^-16^
**Body mass index (kg/m2)**	21.72 (2.66)	30.03 (8.80)	3.88 × 10^-10^
**Smoke (% yes)**	0.33	0.39	0.65
**Alcohol drinking (% yes)**	0.91	0.85	0.45

s.d., standard deviation.

### PBM Isolation

Sixty milliliters of peripheral blood were collected from each subject by a certificated phlebotomist. The fresh blood samples were immediately processed for PBM isolation. First, peripheral blood mononuclear cells (PBMCs) were isolated from whole blood using density gradient centrifugation with Histopaque-1077 (Sigma-Aldrich, USA). Then, PBMs were isolated from PBMCs using Monocyte Isolation Kit II (Miltenyi Biotec Gmbh, Bergisch Glagbach, Germany) according to the manufacturer's protocol. The kit contains a highly optimized antibody mix and blocking reagent to deplete T cells, B cells, and natural killer cells from PBMCs, leaving monocytes untouched and free of surface-bound antibody and beads with minimum disturbance. The purity of PBMs isolated using this method was 86% ± 3%. Genomic DNA was extracted from the freshly isolated PBMs with the AllPrep DNA/RNA/miRNA Universal Kit (Qiagen, Inc., Valencia, CA), following the manufacturer's protocol and stored at −80°C until further use.

### Whole-Genome Sequencing Assay

Genomic DNA samples extracted from the whole blood were sequenced to 30x coverage on Illumina HiSeq X-Ten with 150 bp paired-end reads. Data quality check was performed on the Illumina sequencing analysis viewer (SAV). Sequence reads were trimmed using Cutadapt (version 1.11) (Martin, 2011), aligned to GRCh37 (hg19) using BWA-MEM (version 0.7.12-r1039) (Li and Durbin, 2010), duplicates marked with Picard (version 1.129, http://picard.sourceforge.net), and coordinates sorted using Samtools (version 1.3) (Li et al., 2009). SNPs were detected by a dual calling strategy using qSNP (Kassahn et al., 2013) and GATK HaplotypeCaller (McKenna et al., 2010). Variants were annotated with Ensembl v75 gene feature information. Variants were considered “called” and used in the subsequent analysis if they passed the following filters: a minimum read depth of eight reads in each dataset; at least four reads containing the variant where the variant was identified on both strands and not within the first or last five bases. Variants that did not pass these filters were considered “low evidence”.

### Epigenome-Wide DNA Methylation Assay and Data Processing

Epigenome-wide DNA methylation profiles were determined by reduced representation bisulfite sequencing (RRBS) according to previously published protocols (Meissner et al., 2005). Briefly, 100 ng genomic DNA from PBMs were digested overnight with MspI restriction enzyme (Thermo Scientific, USA) and then subjected to library construction using the NEXTflex Bisulfite-Seq Library Prep Kit and NEXTflex Bisulfite-Seq Barcodes (BioO Scientific, USA) with a modification of bead size selection to capture MspI fragments of 40–220 bp size. The resulting libraries were bisulfite converted by the EZ DNA Methylation-Gold kit (Zymo Research Corp, USA) and amplified by 20 cycles of PCR using the NEXTflex Bisulfite-Seq U+PCR Master Mix and NEXTflex Primer Mix (BioO Scientific, USA). The bisulfite conversion rate of the EZ DNA Methylation-Gold Kit is ≥99% according to the manufacturer's specifications. Different adaptors were used for multiplexing samples into one lane. Library concentrations and quality were measured by Qubit ds DNA HS Assay kit (Life Technologies, USA) and Agilent Bioanalyzer (Agilent, USA). Purified and quantified libraries were pooled at six samples per lane for sequencing and read by 1 × 50 bp on Illumina HiSeq3000. Data quality check was done on Illumina SAV. De-multiplexing was performed with Illumina Bcl2fastq2 v2.17 program and standard FASTQ files were trimmed with Cutadapt v1.3 (Martin, 2011). The trimmed reads were mapped to the human reference genome (hg19) and converted to methylation values on the 0–1 scale using Bismark v0.10.0 (Krueger and Andrews, 2011). Only CpG sites with ≥3-fold coverage in at least 30 subjects in each BMD group were included in the subsequent analysis.

### Control for Potential Cell Admixture

As is true for most tissues, the PBM contains a mixture of several subtypes of cells (Wong et al., 2011). This may cause false positives in association analysis if 1) the methylation pattern of subcell types differs and 2) the relative abundance of cell types is correlated with the outcome variable of interest (Houseman et al., 2012). We adopted a principal component (PC) analysis approach to alleviating the risk of false positive discoveries (Sun et al., 2010; Liu et al., 2013; McClay et al., 2015). The underlying assumption is that subjects with similar cell type compositions will have more similar multi-locus methylation patterns and these patterns can be captured by the PCs. By including the PCs of methylation data as covariates in the association analysis, the potential effects of cell mixture can be regressed out (Sun et al., 2010; Liu et al., 2013; McClay et al., 2015). Similarly, the PC could also help adjust the batch effect and other unmeasured confounders (Price and Robinson, 2018). To obtain the PCs, the methylation data were first normalized by the inverse quantile transformation of the ranked values, which is a robust approach that can reduce the effect of outliers (Wright et al., 2014). Based on the variance explained (**Figure S1**, **Table S1**), the first PC, explaining 26.95% of the methylation variance, was included as a covariate in the following regression analysis.

### Epigenome-Wide Association Analysis

Differential methylation analyses were carried out by using R package *methylKit* (Akalin et al., 2012) with the following logistic regression model at each CpG site for subject *i = 1*,…,*n*

$$\log\frac{P_{i}}{1-P_{i}}=\;\beta_{0}+\beta_{1}*X_{i\;}+\sum_{j=1}^{K}\alpha_{ji\;}\;\times \;covariate_{k,i}$$

where *n* is the total number of subjects, *P_i_* is the proportion of methylated cytosines, *X_i_* denotes the BMD group (= 0 for subjects in the low BMD group and = 1 for subjects in the high BMD group), *β*_0_ denotes the log odds of the control group, and *β*_1_ denotes the log odds ratio between the high and low BMD groups. Covariates in the model included age, body mass index, drinking status, smoking status, and 1^st^ PC of methylation. We include the PC for methylation for batch effect and cell-type adjustment. The *p*-values for testing the null hypothesis *H*_0_: β_1__=_ 0 were adjusted by Bonferroni correction to account for multiple testing problems. We performed simulation analyses to calculate the power of detecting methylation difference using logistic regression at different settings of mean sequencing coverage and probability of methylation in the low BMD group (details see **Supplementary File Part 1**).

### mQTL Analysis

Autosomal analysis was applied to 9,265,832 SNPs with a minor allele frequency > 0.01, genotype hard call rate > 0.95, and Hardy–Weinberg *p* > 1 × 10^−6^. The first PC of SNPs, which explains 46% of the variance (**Figure S2**, **Table S2**), was included as a covariate in the association analysis to control for ancestry and population stratification (Price et al., 2006). To increase the computation speed, we split the SNP and methylation data by chromosome and performed association analysis for each SNP-DMC pair on the same chromosome to identify both *cis*- and *trans*-mQTLs using R package *matrixEQTL*. The SNP was defined *cis* if the distance between SNP and CpG was <1 Mb and *trans* otherwise. *p*-values were corrected by the Bonferroni approach using the total number of tests of *cis* and *trans* effects (**Table S3**) separately to account for the multiple testing problem. SNPs that have a significant (Bonferroni adjusted *p*-value ≤ 0.05) association with DMCs were defined as mQTLs.

### MR Analysis

The DMCs were further analyzed by MR to estimate the potential mediation effects of DNA methylation on BMD. Considering the lack of power for detecting association due to the limited sample size (N = 118) of our study cohort, we undertook a two-sample MR (2SMR) approach with estimated effects between genetic variants and BMD from published studies (Burgess et al., 2015). We used DMCs as the exposure and its associated mQTLs as the instrument variables (IVs). The effects of mQTLs on BMD variation were extracted from the GWAS summary statistics for femoral neck BMD from the GEnetic Factors for OSteoporosis Consortium 2015 data release (Zheng et al., 2015). The mQTL SNPs were further pruned by linkage disequilibrium (LD) correlation. Only SNPs with low LD (*r* ^2^ < 0.2) were retained to ensure the independence of IVs. If there was only one remaining SNP, we used the Wald ratio test to assess the causal effect of DNA methylation. If there were two or more remaining SNPs, we used inverse-variance weighted (IVW) regression (Burgess et al., 2013) along with sensitivity analysis by Egger's regression (Bowden et al., 2015) to assess the causal relationship. We performed the MR analysis iteratively through all the DMCs. The DMCs that achieved FDR corrected significance level in the Wald ratio test or IVW regression were defined as mediator DMCs, i.e. methylation at these CpG sites with mediation effects for genetic influences on BMD. The 2SMR was implemented by R package MR-Base (Hemani et al., 2018).

We also performed reverse MR analysis to evaluate the potential reverse causation (i.e. BMD causes altered DNA methylation at the CpG site of interest) for the mediator DMCs detected in the MR analysis. Because of the limited power to identify SNPs significantly associated with BMD using our study cohort here, the IVs for this analysis were identified with relevant GWAS for BMD reported on the NHGRI-EBI GWAS catalog (MacArthur et al., 2017).

### Fine Mapping

In 2SMR, a set of SNPs that are significantly associated with methylation at a CpG site was required to estimate the causal effect of the methylation. To distinguish the genetic variants with a high probability of causality among all genetic variant associated with a DMC, we performed fine mapping analysis with PAINTOR software (Kichaev et al., 2014; Kichaev et al., 2017). The major advantage of the PAINTOR algorithm is that it incorporates functional annotation information of the genetic variant with the association and LD information to calculate a posterior probability for each SNP to be causal across a locus. Previous simulations have shown that compared to other fine-mapping approaches, PAINTOR has much higher accuracy to select the causal SNPs with annotation information available (Kichaev et al., 2014). LD matrix of the pairwise correlations between SNPs was calculated with PLINK software using our own sample of 118 Caucasian women. Functional annotation included in the analysis were gene elements form GenCode (eight annotations) and chromatin states of monocytes computed from the 15-state hidden Markov model built by NIH Roadmap Epigenomics Project (Roadmap Epigenomics Consortium et al., 2015). The annotation files were downloaded from the PAINTOR website (https://github.com/gkichaev/PAINTOR_V3.0/wiki/2b.-Overlapping-annotations). We set the maximum number of causal variants in a locus to be two based on our computational capability. The mQTLs that have the largest posterior probability as computed by the PAINTOR algorithm were defined as causal mQTLs.

### Test of Pleiotropy Effect

The observation of the mediation effect of DNA methylation in the 2SMR analysis does not necessarily mean that both DNA methylation and BMD are affected by the same causal variant identified by fine mapping (pleiotropy effect). It is possible that different SNPs that are in high LD causally affect DNA methylation and BMD, respectively (linkage effect, **Figure 1B**). Hence, for mediator DMCs, we tested the heterogeneity in the *b_xy_* values estimated for multiple SNPs in the *cis*-eQTL region using the heterogeneity in dependent instruments (HEIDI) method to distinguish pleiotropy from linkage with multiple SNPs in *cis*-region. Under the null hypothesis of the HEIDI test which assumes pleiotropy, the estimated effect size of DNA methylation on BMD (*b_xy_*) calculated for any SNPs in LD with the causal variant will be identical. Therefore, testing against the null hypothesis is equivalent to testing whether there is heterogeneity in the *b_xy_* values estimated for the causal variant and all other SNPs in the *cis*-mQTL region. In the HEIDI test, we only included SNPs in moderate LD with the causal *cis*-mQTL (0.05 < *r* ^2^ < 0.9) which was inferred by the population in our study. We also removed SNPs in the *cis*-mQTL region with an mQTL *p*-value > 1.6 × 10^−3^ to avoid weak instrumental bias which was the default setting of the SMR software (Zhu et al., 2016). *p*-value threshold was adjusted by Bonferroni correction using the total number of identified causal mQTLs.

### Functional Annotation for DMC, Mediator DMC, and Causal mQTLs

To test for functional enrichment of the DMCs, we annotate them to seven regulatory categories, including three general regulatory categories (genic regions, CpG islands, and binding sites of CTCF) and fore cell-type specific regulatory categories of CD14^+^ monocyte (histone marks, DNaseI signal peak, chromatin states from the NIH Roadmap Epigenomics Project (Roadmap Epigenomics Consortium et al., 2015), and monocyte differentially expressed enhancers from Fantom5 (Lizio et al., 2017)). We mapped each DMC to the annotation categories and recorded overlap at each DMC as a binary variable. To determine whether enrichment occurred more frequently than by chance, we randomly generated 1,000 sets of CpGs (sample size = number of DMCs) from all the input CpGs for differential methylation analysis. For each epigenetic mark, we then calculated the proportion of overlapping CpGs among the DMCs (observed) and that of 1,000 simulated sets of CpGs (expected). We calculated the fold enrichment as observed/expected proportion values and obtained an empirical p value from the distribution of expected proportion values.

We investigated the biological meaning of the DMCs by analyzing the annotations of the nearby genes. We used the Genomic Regions Enrichment of Annotations Tool (GREAT) v3.0.0 to evaluate whether the nearby genes of DMCs are enriched in any gene and human phenotypes ontology terms. To better understand the functional mechanism of the identified causal mQTLs, we annotated each of them to the genic region of specific genes and the regulatory categories as we did for DMC annotation. We use the VEP tool (McLaren et al., 2016) for gene annotation and prediction of their functional consequences. In addition, we used the Core Expression Analysis function in the Ingenuity Pathway Analysis software (IPA, QIAGEN Redwood City, www.qiagen.com/ingenuity) to identify overrepresented canonical pathways, diseases, or disorders and molecular and cellular functions for the nearest genes of causal mQTLs. The experimentally observed molecules and relationships in the IPA Knowledge Base for mammal (humans, mouse, or rat) were used. *p*-values of the right-tailed Fisher exact test were calculated by IPA to assess the enrichment. We also performed gene pathway enrichment analyses using Metascape (Zhou Y. et al., 2019), in which the following ontology sources were used: KEGG Pathway, GO Biological Processes, Reactome Gene Sets, Canonical Pathways and CORUM. All genes in the genome were used as the enrichment background. Terms with a *p*-value < 0.01, a minimum count of 3, and an enrichment factor > 1.5 (the enrichment factor is the ratio between the observed counts and the counts expected by chance) were collected and grouped into clusters based on their membership similarities. The most statistically significant term within a cluster was chosen to represent the cluster (Zhou Y. et al., 2019).

## Results

### Differentially Methylated CpG Sites

A total of 17,462,566 CpG sites were measured in 54 subjects in the low BMD group and 64 subjects from the high BMD group, of which 1,267,919 CpG sites that were measured with adequate sequencing coverage (N ≥ 3) in more than 30 subjects in each BMD group were used for differential methylation analysis. The mean sequencing coverage of the tested CpGs was 46.52 (95% CI: 22.95~62.09) fold. We identified 6,149 DMCs significantly associated with BMD at *Pp* < 3.94 × × 10^-8^ (Bonferroni correction 0.05/1,267,919), including 2,188 DMCs with an absolute difference in methylation values > 0.05 and 693 DMC with absolute difference ≥ 0.1 between the low and high BMD groups (**Table S4** and **Figure S3**). Among the 2,188 DMCs with an absolute difference in methylation values > 0.05, 1,601 (74%) DMCs had mean sequencing coverage >10 and 1,161 (53%) had mean coverage >30 (**Figure S4**). We estimated that our sample size has approximately 80% power to detect a 0.05 gross difference (total difference caused by methylation and other covariates) at the mean coverage of 30-fold (**Figure S5**). We used relatively moderate thresholds for the sequencing coverage and methylation difference in DMC analyses in order to provide a large set of candidates for the downstream analyses.

**Figure 3:**
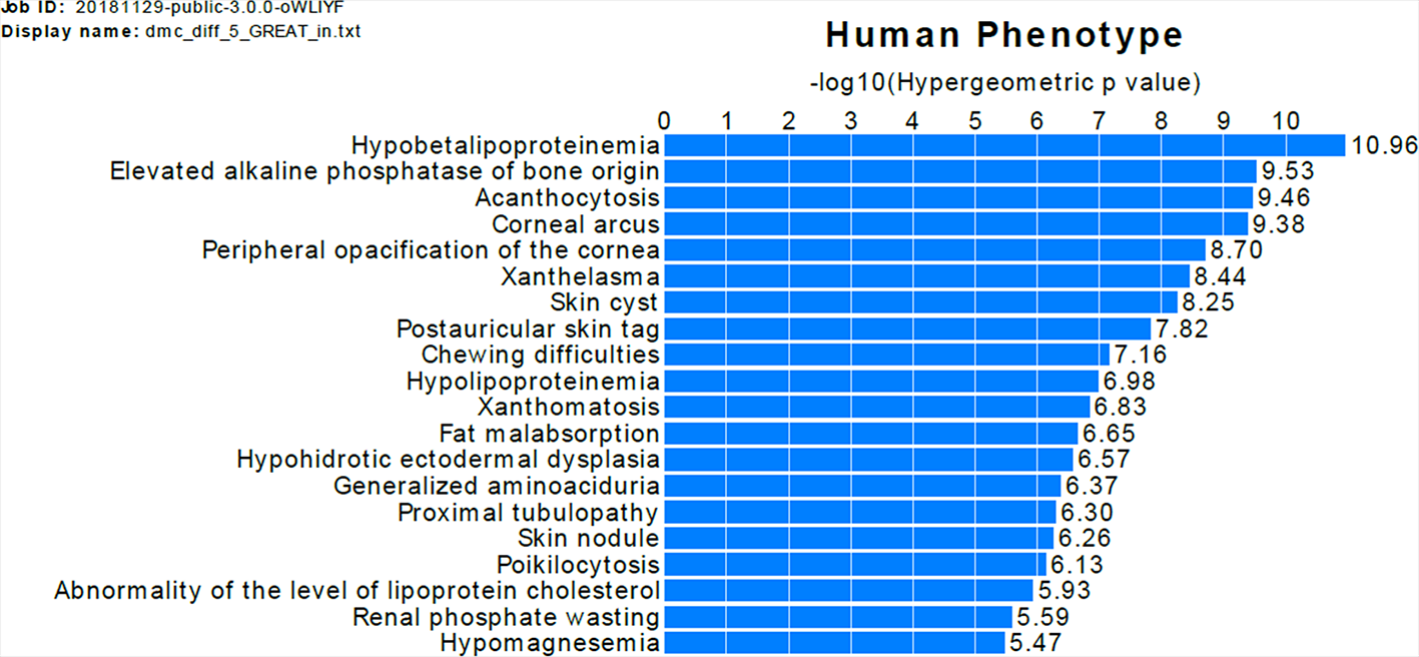
Functional annotation of DMCs with difference >0.05. The bars represent the top 20 categories in the human phenotype ontology enriched genes near the DMCs. The length of the bars corresponds to values on the x-axis, which are hypergeometric (uncorrected) *p*-values.

The GREAT analysis shows that the nearby genes of DMCs with difference >0.05 are enriched in association with a bone-related human phenotype term, *elevated alkaline phosphatase of bone origin* (**Figure 3**). To evaluate whether our findings are consistent with previous studies, we checked the overlap of DMCs with the significant signals in three previous EWAS of OP (Delgado-Calle et al., 2013; Reppe et al., 2017; Morris et al., 2017). For each DMC identified by our study, we found the nearest signals identified in previous EWAS and calculated the distance between the DMC and the EWAS signals (**Tables S5** and **S6**). Twelve DMCs are located within 1,000 bp from the previously identified EWAS signals by Delgado-Calle et al. (Delgado-Calle et al., 2013). The low overlap rate between our results and that of Delgado-Calle's study is likely due to the very limited overlap of the tested CpG sites between the two studies, and the difference in the studied sample cohorts. Specifically, although we tested 1,267,919 CpGs measured by RRBS, only 33,345 (2.6%) of the tested CpGs were mapped to within 1,000 bp from the CpGs measured by the Illumina 27K array, which was used in the study by Delgado-Calle et al. In addition, our study was perform in PBMs from women aged between 20 and 40 years, whereas the study by Delgado-Calle et al. studied trabecular bone specimens from women aged 59–85 years, with 26 had osteoarthritis and 27 had OP fractures.

### Construction of Causal Paths: mQTLs, Mediator DMCs and Causal mQTLs

As we were particularly interested in DMCs where the DNA methylation may mediate the genetic risk for OP, we applied 2SMR analysis on the 6,149 DMCs. First, we identified 115,727 significant *cis*-DMC-mQTL pairs with *Pp* < 1.08 × × 10^-9^ (Bonferroni correction 0.05/46,331,365) and 79,188 significant *trans*-DMC-mQTL pairs with *Pp* < 1.83 × × 10^-11^ (Bonferroni correction 0.05/2,736,230,941), including 125,409 unique mQTLs and 2,618 unique DMCs.

Next, by using mQTLs as the instrumental variables, DNA methylation at DMCs as potential mediators, and BMD groups as the outcome, we found 30 significant mediator DMCs in the 2SMR analysis (**Table 2**), among which 11 mediator DMCs showed a positive relationship between DNA methylation and BMD and 19 showed a negative relationship based on the estimated effect sizes. The minimum absolute differences in methylation values of the mediator DMCs is 1.6% between the high versus low BMD groups, while half of mediator DMCs have methylation difference greater than 5% (**Table 2**).

**Table 2:** Results of Mendelian randomization analysis.

DMC^1^	Method	nSNP	b_MR	se_MR	*p*_MR	b_Egger	se_Egger	*p*_Egger	Top SNP	Top SNP bp	SNP Overlapped Gene	*p*_HEIDI	nSNP_HEIDI	Meth_diff	*p*_DMC
**1:22571385**	IVW	4	0.022	0.004	2.26E-06	0.085	0.051	0.240	rs10917185	22571385	MIR4418	0.341	14	10.600	3.27E-10
**2:31719473**	IVW	2	-0.024	0.007	3.36E-02	NA	NA	NA	rs151081363	31720272	SRD5A2	0.335	14	-8.963	5.93E-31
**4:957285**	IVW	2	0.017	0.005	1.86E-02	NA	NA	NA	rs73211813	975238	DGKQ/SLC26A1	0.543	16	-10.465	4.49E-28
**6:32222711**	IVW	5	-0.030	0.007	3.22E-03	0.162	0.065	0.088	rs454748	32213210	XXbac-BPG154L12.4	0.517	9	-3.770	9.52E-17
**7:1062527**	IVW	4	0.016	0.004	2.41E-02	-0.001	0.032	0.981	rs4388364	1033045	C7orf50	0.506	20	-2.774	5.29E-17
**7:56435427**	IVW	10	0.016	0.003	1.28E-05	-0.019	0.024	0.444	rs3813509	56356143	RP11-700P18.1	0.853	8	-11.186	8.40E-47
**8:58127658**	IVW	9	-0.025	0.003	9.54E-11	0.003	0.041	0.939	rs11786043	58133174	RP11-513O17.2	0.663	18	5.138	1.30E-31
**9:71682281**	IVW	4	0.016	0.004	2.89E-03	-0.025	0.066	0.744	rs10116497	71634393	PRKACG	0.360	20	-9.207	6.99E-54
**9:96362114**	IVW	5	0.017	0.004	3.22E-03	0.003	0.137	0.982	rs7857266	96381765	PHF2	0.376	20	1.662	2.41E-18
**10:42739065**	IVW	6	0.016	0.004	3.36E-02	0.099	0.173	0.596	rs2489684	42862079	RP11-313J2.1	0.298	20	-3.140	1.70E-18
**12:48723325**	IVW	4	-0.017	0.005	2.90E-02	-0.049	0.064	0.519	rs10875744	48498440	SENP1	0.433	9	12.018	1.45E-100
**14:75441795**	IVW	3	0.015	0.004	3.28E-02	-0.111	0.730	0.904	rs35446981	75637351	TMED10	0.228	4	-6.827	5.95E-11
**16:81248716**	IVW	2	-0.019	0.005	4.20E-02	NA	NA	NA	rs12925557	81251149	PKD1L2	NA	NA	-10.155	1.37E-29
**16:89167395**	IVW	3	0.021	0.006	2.91E-02	0.041	0.049	0.554	rs7188200	89167094	ACSF3	0.557	13	7.130	6.11E-24
**17:1944903**	WR	1	-0.056	0.012	2.57E-03	NA	NA	NA	rs76439887	1945201	DPH1	NA	NA	-2.516	2.22E-10
**17:1944905**	WR	1	-0.061	0.013	2.57E-03	NA	NA	NA	rs76439887	1945201	DPH1	NA	NA	-3.052	6.39E-20
**17:42246289**	IVW	2	-0.042	0.006	1.65E-10	NA	NA	NA	rs2526020	42216588	C17orf53	0.451	5	19.333	2.65E-21
**17:44060776**	IVW	44	0.013	0.001	5.94E-37	0.017	0.017	0.322	rs79724577	43463493	MAPT	0.735	5	8.741	2.70E-10
**17:43894548**	IVW	40	0.011	0.001	2.02E-31	0.016	0.006	0.008	rs62054394	43810608	CRHR1	0.312	17	7.835	2.75E-08
**17:43828617**	IVW	41	0.015	0.001	5.59E-35	0.015	0.012	0.203	rs62063779	44054671	ARHGAP27	0.177	20	5.094	1.22E-12
**17:44337590**	IVW	38	-0.022	0.002	3.65E-30	-0.038	0.014	0.011	17:44359021	44359021	ARL17B	0.175	19	-2.539	2.87E-22
**17:44337597**	IVW	42	-0.022	0.002	3.75E-34	-0.052	0.017	0.004	17:44359021	44359021	ARL17B	0.192	20	-2.557	4.01E-22
**17:44337613**	IVW	38	-0.022	0.002	5.54E-30	-0.040	0.013	0.004	17:44359021	44359021	ARL17B	0.175	19	-2.316	7.11E-19
**17:44337604**	IVW	42	-0.022	0.002	2.91E-36	-0.005	0.022	0.819	17:44366572	44366572	ARL17B	0.192	20	-2.308	4.20E-18
**17:44337617**	IVW	40	-0.022	0.002	1.65E-33	-0.020	0.016	0.220	17:44572989	44572989	RP11-995C19.2	0.176	19	-2.630	5.94E-21
**17:44337622**	IVW	42	-0.023	0.002	2.91E-36	-0.013	0.021	0.545	17:44572989	44572989	RP11-995C19.2	0.176	19	-2.637	2.50E-20
**17:80053590**	IVW	6	0.013	0.003	1.30E-03	0.000	0.021	0.984	rs59251877	80056498	FASN	0.004	7	8.662	1.50E-23
**17:80086159**	IVW	7	0.014	0.003	7.07E-04	0.004	0.017	0.831	rs59251877	80056498	FASN	0.004	8	3.921	5.50E-11
**21:46677414**	IVW	5	0.013	0.004	4.22E-02	-0.013	0.030	0.688	rs28622522	46676599	LINC00334	0.403	20	-2.369	1.53E-09
**22:46504167**	IVW	2	-0.026	0.007	1.05E-02	NA	NA	NA	rs12170325	46502870	FLJ27365	0.880	4	-4.806	2.14E-11

^1^Format of DMC, chromosome: position.

nSNP, number of SNPs; b, effect size; se, standard error; p, p-value; bp, base pair position; Meth_diff, difference in mean methylation level between BMD groups (high BMD group minus low BMD group). NA stands for not applicable. NA occurs because the number of associated SNPs (nSNP) is less than three for the Egger regression statistics and no linkage SNP found to be tested for HEIDI test.

To evaluate the potential reverse causation between BMD and methylation at the 30 DMCs, we also carried out reverse 2SMR analysis by using SNPs associated with BMD as the instrumental variables, BMD as the potential mediators, and DNA methylation at DMCs as the outcome. None of the 30 mediator DMCs showed significant evidence of reverse causality after multiple testing correction (**Table S7**). Two DMCs (Chr16:89167395 and Chr2:31719473) reached the nominal significance level (0.05) for both the two-stage least square (2SLS) method with multiple linear regression and 2SLS with logistic regression (**Table S8**), which further supports the existence of mediation effect at these CpG sites.

The 30 mediator DMCs were fine mapped to 25 causal mQTLs (**Table 2**). None of the causal mQTLs has a HEIDI test *p*-value past the threshold 0.001 (0.05/30) after Bonferroni adjustment, suggesting it is unlikely that the SNPs in LD with these causal mQTLs affect BMD.

### Biological Significance of the Causal Paths

The mediator DMC–causal mQTL pairs were mapped to 25 unique genes (the nearest genes of causal mQTLs), of which eight genes have been associated with bone-related phenotype in previous GWAS studies (**Table 3**). Three causal mQTLs are in high LD with BMD-associated SNPs reported in GWAS catalog: rs2526020 (in LD with rs227584, *r* ^2^ = 0.8913), rs62054394 (in LD with rs1864325, *r* ^2^ = 1), rs62063779 (in LD with rs1864325, *r* ^2^ = 1). Four causal mQTLs were shared by more than one mediator DMC (rs76439887, 17:44359021, 17:44572989, and rs59251877), which may point to the location of some harbor genes. By overlapping the causal mQTLs and the mediator CpGs with regulatory categories, we found that 10 out of 25 causal mQTLs and 25 out of 30 mediator DMCs reside within cell type-specific histone mark peaks, enhancers, promoters, promoter flanking regions and CTCF binding sites (**Tables S9** and **S10**), supporting the regulatory potential of these loci.

**Table 3:** Gene annotation of causal mQTLs.

DMC	Causal mQTLs	mQTL bp	Causal mQTLs Overlapped Gene	Gene Type	Traits	GwasCatalog Study ID	PMID
**1:22571385**	rs10917185	22571385	MIR4418	miRNA	BMD	GCST001482	22504420
					BMD (total hip)	GCST006143	29883787
					Heel BMD	GCST006433	30048462
					Heel BMD	GCST006979	30598549
					Heel BMD	GCST007066	30595370
					Lumbar spine BMD (integral)	GCST007015	27476799
					Lumbar spine BMD (trabecular)	GCST007014	27476799
					Total body BMD	GCST005348	29304378
**2:31719473**	rs151081363	31720272	SRD5A2	Processed transcript			
**4:957285**	rs73211813	975238	DGKQ	Protein coding	Heel BMD	GCST006433	30048462
**4:957285**	rs73211813	975238	SLC26A1	Protein coding			
**6:32222711**	rs454748	32213210	XXbac-BPG154L12.4	Antisense			
**7:1062527**	rs4388364	1033045	C7orf50	Protein coding	LDL measurement	GCST006612	30275531
**7:56435427**	rs3813509	56356143	RP11-700P18.1	Pseudogene			
**8:58127658**	rs11786043	58133174	RP11-513O17.2	lincRNA			
**9:71682281**	rs10116497	71634393	PRKACG	Protein coding	Blood pressure/cancer	GCST007087/GCST005275	30595370/29299148
**9:96362114**	rs7857266	96381765	PHF2	Protein coding	Heel BMD	GCST006288	28869591
					Heel BMD	GCST006433	30048462
					Heel BMD	GCST006979	30598549
						GCST007066	30595370
**10:42739065**	rs2489684	42862079	RP11-313J2.1	Pseudogene			
**12:48723325**	rs10875744	48498440	SENP1	Protein coding			
**14:75441795**	rs35446981	75637351	TMED10	Protein coding	Heel BMD	GCST006433	30048462
**16:81248716**	rs12925557	81251149	PKD1L2	Polymorphic pseudogene			
**16:89167395**	rs7188200	89167094	ACSF3	Protein coding			
**17:1944903**	rs76439887	1945201	DPH1	Protein coding			
**17:1944905**	rs76439887	1945201	DPH1	Protein coding			
**17:42246289**	rs2526020	42216588	C17orf53	Protein coding	BMD	GCST000297	19079262
					BMD	GCST001482	22504420
**17:44060776**	rs79724577	43463493	ARHGAP27	Protein coding	Heel BMD	GCST006433	30048462
**17:43894548**	rs62054394	43810608	CRHR1	Protein coding	BMD (spine)	GCST000494	
					BMD (hip)	GCST000495	
					Heel BMD	GCST006433	
					Heel BMD	GCST006979	
					Heel BMD	GCST007066	
**17:43828617**	rs62063779	44054671	MAPT	Protein coding	BMD	GCST001482	22504420
					Heel BMD	GCST006433	30048462
**17:44337590**	17:44359021	44359021	ARL17B	Protein coding			
**17:44337597**	17:44359021	44359021	ARL17B	Protein coding			
**17:44337613**	17:44359021	44359021	ARL17B	Protein coding			
**17:44337604**	17:44366572	44366572	ARL17B	Protein coding			
**17:44337617**	17:44572989	44572989	RP11-995C19.2	Pseudogene			
**17:44337622**	17:44572989	44572989	RP11-995C19.2	Pseudogene			
**17:80053590**	rs59251877	80056498	FASN	Protein coding			
**17:80086159**	rs59251877	80056498	FASN	Protein coding			
**21:46677414**	rs28622522	46676599	LINC00334	lincRNA			
**22:46504167**	rs12170325	46502870	FLJ27365	Protein coding			

bp, base pair position.

**Figure 4:**
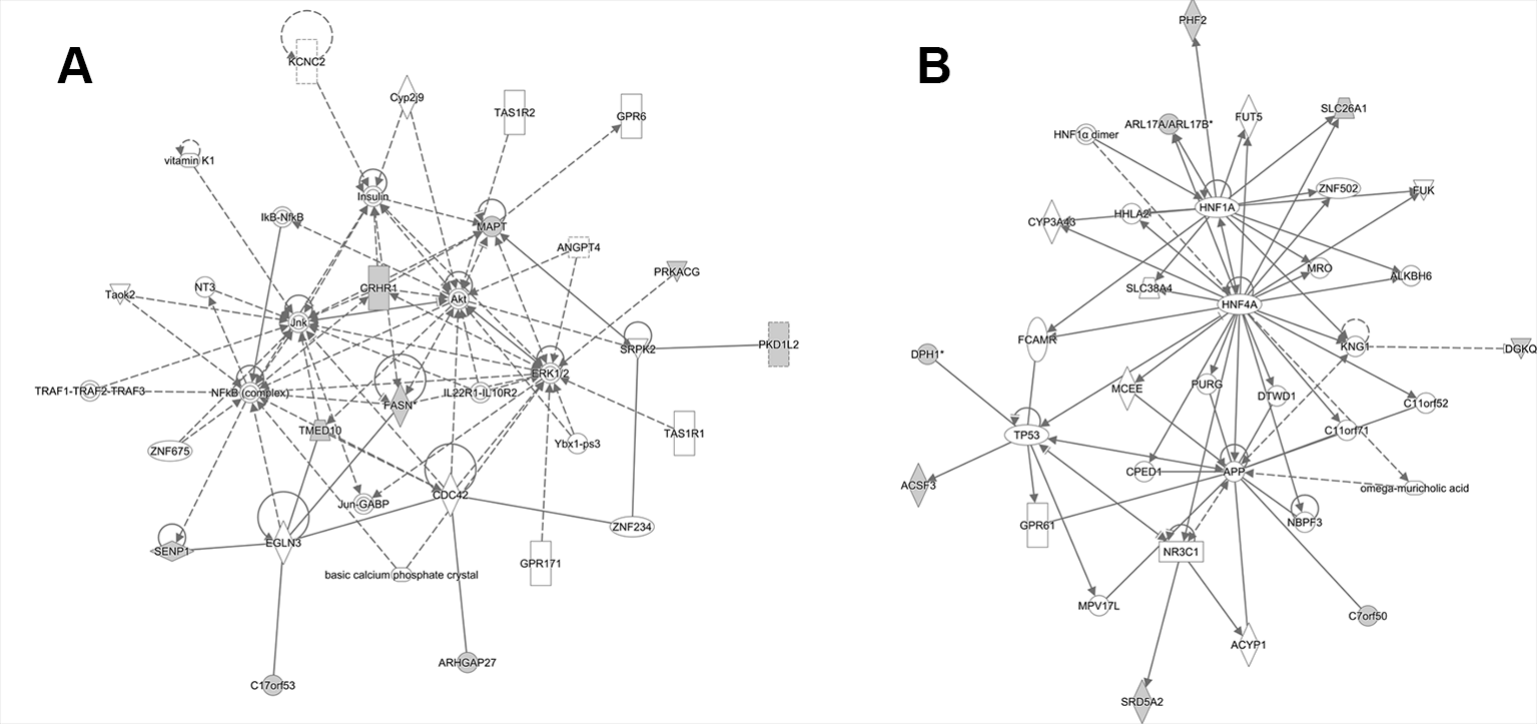
Two predicted nondirectional interaction map of causal mQTL annotated gene by IPA. **(A)** Top mapped disease or functions: cell-to-cell signaling and interaction, cellular assembly, and organization and cellular function and maintenance. **(B)** Top mapped disease or functions: endocrine system disorders, gastrointestinal disease, hereditary disorder. The shaded nodes are gene identified by our analysis. And others are genes/molecules from the Knowledge Base added to the network to fill or join areas lacking connectivity by IPA. Highly interconnected networks are likely to represent significant biological function. For detailed legend see IPA Legend page: http://qiagen.force.com/KnowledgeBase/articles/BasicTechnicalQA/Legend.

Using IPA software, we identified the top canonical pathways related to the causal mQTLs, shown in **Table 4**. In our result, we didn't find the most well-known signaling pathways related to bone metabolism, like the Wnt and BMPs (bone morphogenetic proteins) (Shahi et al., 2017). However, it has been established that OP and cardiovascular disease are functionally interwoven and share some common risk factors like hyperlipidemia (Parhami et al., 2000). The identification of ‘Fatty Acid Biosynthesis Initiation II’ in the top canonical pathways, ‘Lipid Metabolism’ in the top molecular and cellular functions and ‘Cardiovascular disease’ in the top diseases and disorders (**Table 4**) may indicate that lipid metabolism has more important effects on BMD than previously understood. We also performed gene pathway enrichment analysis using Metascape (Zhou Y. et al., 2019). A GO biological process term related to lipid metabolism (steroid biosynthetic process) showed significant enrichment (*p*-value = 1.45 × × 10^-4^) in genes mapped to causal mQTLs which further supported our findings by IPA (**Figure S6** and **Table S13**). Finally, we provided a network graph for the genes near the identified causal mQTLs (**Figure 4**), which elucidates the interaction between the identified genes in more detail and may facilitate researchers to discover new drug targets or disease mechanisms.

**Table 4:** Canonical pathways, diseases, and molecular and cellular functions significantly enriched in genes mapped to causal mQTLs.

Top Canonical Pathways	*p*-value^1^	Overlap^2^
Amyloid processing	6.79E-04	3.9% 2/51
Palmitate biosynthesis	1.52E-03	50.0% 1/2
Fatty acid biosynthesis initiation II	1.52E-03	50.0% 1/2
Gas signaling	2.94E-03	1.9% 2/107
CDK5 signaling	3.00E-03	1.9% 2/108
Diseases and Disorders	***p*-value range** ^3^	**#Molecules** ^4^
Neurological Disease	4.90E-02–5.29E-04	5
Organismal Injury and Abnormalities	4.76E-02–5.29E-04	17
Cancer	4.69E-02–7.60E-04	17
Cardiovascular Disease	3.52E-02–7.60E-04	1
Connective Tissue Disorders	4.76E-02–7.60E-04	5
Molecular and Cellular Functions	***p*-value range** ^3^	**#Molecules** ^4^
Lipid Metabolism	4.40E-02–3.16E-04	6
Small Molecule Biochemistry	4.40E-02–3.16E-04	7
Nucleic Acid Metabolism	1.21E-02–3.78E-04	5
Cell Morphology	4.76E-02–7.60E-04	5
Cell-To-Cell Signaling and Interaction	4.39E-02–7.60E-04	3

^1^The Fisher's exact test *p*-value indicating the significance of enrichment of the causal mQTL genes in the pathway.

^2^In a given pathway, the overlap is calculated as the number of causal mQTL genes enriched in the pathway divided by the total number of genes in that pathway.

^3^The Fisher's exact p-value indicating the range of enrichment in the subcategories of each disease/function.

^4^The number of genes that are associated with each disease/function.

## Discussion

In this article, we developed a framework for evaluating the causal effect of DNA methylation on a complex trait. This framework combines the MR method with fine mapping and colocalization so that it can distinguish causal relationship from reverse causality, linkage and horizontal pleiotropy (**Figure 1**). Using this framework, we successfully identified 30 causal pathways from genetic variant to BMD phenotype mediated by DNA methylation. Among the 25 corresponding causal genes that we identified, eight genes (*MIR4418, TMED10, C17orf53, CRHR1, ARHGAP27, MAPT, DGKQ, and PHF2*) have been reported to be associated with BMD in previous GWAS (**Table 3**).

The *CRHR1* gene was first reported to be significantly associated with BMD in a large-scale meta-analysis of GWAS in 19,195 adults of Northern European descent (Rivadeneira et al., 2009), and this association was also replicated in East Asian populations (Styrkarsdottir et al., 2010). *CRHR1* encodes a G-protein coupled receptor that binds with the neuropeptides of the corticotropin-releasing hormone family, a major regulator of the hypothalamic-pituitary-adrenal pathway with important effects on bone turnover (Ralston and Uitterlinden, 2010). We identified six causal CpGs located in a CpG island (chr17:44337401-44337926) near the 5'UTR region of the *CRHR1* gene, of which five are hypo-methylated in the low BMD group and one is hypermethylated. The hypomethylation in the promoter region of *CRHR1* has the potential role of increasing *CRHR1* expression as was identified in a case-control study of panic disorder (Schartner et al., 2017). In addition, depression, anxiety, and stress have been found to be negatively correlated with BMD (Erez et al., 2012). These findings suggested a potential epigenetic regulatory mechanism between hypomethylation of *CRHR1* promoter and low BMD through the regulation of *CRHR1* gene expression.

*C17orf53* gene was associated with BMD and fracture risk in a large genome-wide meta-analysis study, including 17 GWAS and 32,961 individuals of European and East Asian ancestry (Estrada et al., 2012). A recent study investigated the molecular and cellular functions of a specific BMD-associated SNP (rs227584) in *C17orf53* gene using osteoblastic cells and demonstrated that the SNP rs227584 may alter substrate‐kinase interaction between protein C17orf53 and NEK2 and subsequently regulate osteoblast growth and activity (Zhou X. et al., 2019). Our findings complement the previous results and provide further supporting evidence for the significance of *C17orf53* gene on regulating BMD variation and OP risk.

The analytical framework we developed in this study has several prominent strengths. First, we used multiple IVs in the MR analysis, which can offset the low power problem induced by small sample size (Palmer et al., 2012) and allow for a causal estimate of greater precision than the estimate from any of the individual variants (Pierce et al., 2011).

Secondly, using multiple independent IVs made it possible to perform sensitivity analysis to assess the bias of the MR results when the IV assumptions are prone to be violated. Horizontal pleiotropy is one of the major potential violations and occurs when the variant influences other traits outside of the pathway of the exposure of interest and has an impact on the target outcome or when the variant has a direct effect on the target outcome. The assumption of no horizontal pleiotropy in classic MR methods is hard to establish unequivocally. Thus, we performed MR analysis by IVW regression alongside the sensitivity analysis by Egger regression to allow for the potential existence of horizontal pleiotropy. IVW will return an unbiased estimate if the horizontal pleiotropy is balanced, that is, the deviation from the mean estimate is independent of all other effects. In addition, Egger regression further relaxes the assumption to the InSIDE (Instrument Strength Independent of Direct Effect) condition (Bowden et al., 2015), where the instrument-exposure and pleiotropic effects are uncorrelated. The method returns an unbiased causal effect under the InSIDE condition even if the vertical pleiotropy assumption is violated for all SNPs (Hemani et al., 2018). We identified mediator DMCs primarily based on the test statistics of IVW regression because the Egger regression has very limited power to detect causal effects compared with IVW regression (Bowden et al., 2015). The Egger regression *p*-values of some mediator DMCs are large, suggesting that the IVW estimate might be biased. However, the causal effects were also assessed by IVW with fixed effects, IVW with multiplicative random effect (IVW-MRE), weighted median and maximum likelihood method. The estimates from other MR methods were consistent with those from the IVW regression in general (**Table S11**). Moreover, both the IVW-MRE and weighted median estimates are robust to the existence of horizontal pleiotropy (Bowden et al., 2016; Bowden et al., 2017). Thus, we are confident about the estimated effects and the statistical inference of our results. Further research could be done under our analytical framework substituting IVW regression with the recently published MR-PRESSO method that can test and correct the bias caused by horizontal pleiotropy (Verbanck et al., 2018).

Thirdly, our approach has increased power to detect genetic heritability that could be missing in traditional GWAS. The causal mQTLs annotated to those genes associated with bone-related phenotypes in our analysis do not show genome-wide significance in association with BMD (**Table S12**). They were not reported to be associated with BMD as we searched in the GWAS catalog database. This phenomenon can be explained by the biological interaction of genetic and epigenetic modification. As the causal mQTLs can only influence the trait *via* the mediation effect of methylation, the causal effect of mQTLs on BMD might be offset and not observed in GWAS without controlling for the mediation effect of DNA methylation. The 2SMR method alone can only give us inferences about the mediator CpGs, not the causal variants. Our analytical framework further incorporates fine mapping of mQTLs and uncovered heritable genetic variants contributing to BMD that are invisible to conventional GWAS by leverage methylation data.

It should be noted that some of the identified DMCs should be treated with caution because of relatively low sequencing coverage, zero-inflated methylation data, and/or relatively high over-dispersion levels (**Tables S4** and **S14**). Low sequencing coverage affects both sensitivity and specificity in DMC identification depending on the sample size (Ziller et al., 2015). In addition, an excessive number of zeros in the data might cause extra variance that is difficult for the logistic regression to handle (Sweeney et al., 2014). Although a few statistical approaches (e.g. the zero-inflated binomial model (Hall, 2000)) haven proposed for handling zero-inflated data, however, these approaches have not yet been systemically evaluated for the analysis of DNA methylation data. A comprehensive simulation study is necessary to benchmark the performance of these approaches in DNA methylation analysis, which might be a future extension for us to track. Over-dispersion is another problem likely to happen fitting the methylation data with logistic regression. It refers to a problem of more variability in the data than assumed by the distribution and usually occurs when the observed data does not come from *iid* (independent and identically distributed) distribution (Hilbe, 2009). It is realistic to model the observed RRBS data *y_i_* at a CpG site by binomial distribution with subject-specific parameter *N_i_* and group-specific parameter *p*, where *N_i_* represents the sequencing coverage of the subject *i* and *p* represents the probability of methylation. However, the logistic regression method assumes *N_i_* were the same for all subjects, thus may produce inflated association signals for differential methylation analysis (Klein and Hebestreit, 2016). We estimated the over-dispersion scalar for a DMC as the ratio of the observed over the expected variance of methylation value (**Tables S4** and **S14**). The level of over-dispersion induced inflation of association in differential methylation analysis has not been systematically investigated and is out of the scope of this study. *MethylKit* updated its user's guide very recently and is expected to bring up a function for over-dispersion correction by McCullagh and Nelder's approach (McCullagh, 2019). This new feature could benefit future studies for differential methylation analysis by logistic regression.

The scope of this analysis is to identify the causal paths that are most likely to have a real biological effect on BMD. The causal effect of genetic variants or mediation effect of CpGs should be validated with functional experiments instead of a purely statistical approach. We hope that the identified causal mQTL and mediation CpGs can help to narrow down and prioritize the gene list for potential follow-up biological studies for further validating whether they truly have any functional or pathological significance.

In summary, we demonstrated the value of integration of MR, fine mapping and colocalization analyses in uncovering the causal mechanisms across multi-omics. This approach allowed us to identify CpGs that have mediation influences on genetic variants as well as the proximal causal SNPs that affect the methylation levels at those CpGs. Our results provided novel insights into the genetic and epigenetic mechanisms underlying OP. The identified causal mQTLs and mediation CpGs warrant further functional studies in cell and animal models.

## Data Availability Statement

The genomic sequencing data generated for this study can be found in the NIH dbGaP repository. The dbGaP accession number is phs001960.v1.p1.

## Ethics Statement

The studies involving human participants were reviewed and approved by Tulane University School of Public Health and Tropical Medicine in the United States (IRB #: 10-184088). The patients/participants provided their written informed consent to participate in this study.

## Author Contributions

FY and HS conceived the study. FY, CQ and CX analyzed the data. L-JZ and LW recruited subjects and collected samples. CQ coordinated and performed DNA extraction experiments for sequencing. QT provide database support. FY prepared the manuscript. HS and H-WD contributed to manuscript preparation and feedback. All authors read and approved the manuscript.

## Funding

This study was partially supported or benefited by grants from the National Institutes of Health [R01AR069055, R01MH104680, R01AR059781, R01AG061917, U19AG055373, and P20GM109036], and the Edward G. Schlieder Endowment and the Drs. W. C. Tsai and P. T. Kung Professorship in Biostatistics from Tulane University.

## Conflict of Interest

The authors declare that the research was conducted in the absence of any commercial or financial relationships that could be construed as a potential conflict of interest.

## References

[B1] Akalin A. Kormaksson M. Li S. Garrett-Bakelman F. E. Figueroa M. E. Melnick A. (2012). MethylKit: a comprehensive R package for the analysis of genome-wide DNA methylation profiles. Genome Biol. 13 (10), R87. 10.1186/gb-2012-13-10-r87 PMC3491415 23034086

[B2] Al-Barghouthi B. M. Farber C. R. (2019). Dissecting the genetics of osteoporosis using systems approaches. Trends In Genet. 35 (1), 55–67. 10.1016/j.tig.2018.10.004 30470485 PMC6309493

[B3] Arden N. K. Baker J. Hogg C. Baan K. Spector T. D. (2009). The heritability of bone mineral density, ultrasound of the calcaneus and hip axis length: a study of postmenopausal twins. J. Bone Miner Res. 11 (4), 530–534. 10.1002/jbmr.5650110414 8992884

[B4] Bowden J. Smith G. D. Burgess S. (2015). Mendelian randomization with invalid instruments: effect estimation and bias detection through Egger regression. Int. J. Epidemiol. 44 (2), 512–525. 10.1093/ije/dyv080 26050253 PMC4469799

[B5] Bowden J. Davey Smith G. Haycock P. C. Burgess S. (2016). Consistent estimation in mendelian randomization with some invalid instruments using a weighted median estimator. Genet. Epidemiol. 40 (4), 304–314. 10.1002/gepi.21965 27061298 PMC4849733

[B6] Bowden J. Del Greco M. F. Minelli C. Davey Smith G. Sheehan N. Thompson J. (2017). A framework for the investigation of pleiotropy in two-sample summary data Mendelian randomization. Stat. Med. 36 (11), 1783–1802. 10.1002/sim.7221 28114746 PMC5434863

[B7] Brent Richards J. Zheng H. F. Spector T. D. (2012). Genetics of osteoporosis from genome-wide association studies: advances and challenges. Nat. Rev. Genet. 13 (8), 576–588. 10.1038/nrg3228 22805710

[B8] Burgess S. Butterworth A. Thompson S. G. (2013). Mendelian randomization analysis with multiple genetic variants using summarized data. Genet. Epidemiol. 30 (7), 543–552. 10.1002/gepi.21758 PMC4377079 24114802

[B9] Burgess S. Scott R. A. Timpson N. J. Smith G. D. Thompson S. G. (2015). Using published data in Mendelian randomization: a blueprint for efficient identification of causal risk factors. Eur. J. Epidemiol. 35 (11), 1880–1906. 10.1007/s10654-015-0011-z PMC4516908 25773750

[B10] Burgess S. Dudbridge F. Thompson S. G. (2016). Combining information on multiple instrumental variables in Mendelian randomization: comparison of allele score and summarized data methods. Stat. Med. 35 (11), 1880–1906. 10.1002/sim.6835 26661904 PMC4832315

[B11] Cao Z. Moore B. T. Wang Y. Peng X. H. Lappe J. M. Recker R. R. (2014). MiR-422a as a potential cellular microRNA biomarker for postmenopausal osteoporosis. PloS One. 9 (5), e97098. 10.1371/journal.pone.0097098 24820117 PMC4018259

[B12] Davey Smith G. Ebrahim S. (2003). “Mendelian randomization”: can genetic epidemiology contribute to understanding environmental determinants of disease? Int. J. Epidemiol. 32 (1), 1–22. 10.1093/ije/dyg070 12689998

[B13] Davey Smith G. Hemani G. (2014). Mendelian randomization: genetic anchors for causal inference in epidemiological studies. Hum. Mol. Genet. 23(R1), R89–R98. 10.1093/hmg/ddu328 25064373 PMC4170722

[B14] Delgado-Calle J. Fernández A. F. Sainz J. Zarrabeitia M. T. Sañudo C. García-Renedo R. (2013). Genome-wide profiling of bone reveals differentially methylated regions in osteoporosis and osteoarthritis. Arthritis Rheumatol. 65 (1), 197–205. 10.1002/art.37753 23124911

[B15] Deng H. W. Shen H. Xu F. H. Deng H. Y. Conway T. Zhang H. T. (2002). Tests of linkage and/or association of genes for vitamin D receptor, osteocalcin, and parathyroid hormone with bone mineral density. J. Bone Miner Res. 17 (4), 678–686. 10.1359/jbmr.2002.17.4.678 11918225

[B16] Deng F. Y. Zhu W. Zeng Y. Zhang J. G. Yu N. Liu Y. Z. (2014). Is GSN significant for hip BMD in female Caucasians? Bone. 63, 69–75. 10.1016/j.bone.2014.02.015 24607942 PMC4127973

[B17] Du Y. Zhao L.-J. Xu Q. Wu K. H. Deng H.-W. (2017). Socioeconomic status and bone mineral density in adults by race/ethnicity and gender: the Louisiana osteoporosis study. Osteoporos Int. 28 (5), 1699–1709. 10.1007/s00198-017-3951-1 28236128

[B18] Erez H. B. Weller A. Vaisman N. Kreitler S. (2012). The relationship of depression, anxiety and stress with low bone mineral density in post-menopausal women. Arch. Osteoporos. 7 (1–2), 247–255. 10.1007/s11657-012-0105-0 23095987

[B19] Estrada K. Styrkarsdottir U. Evangelou E. Hsu Y. H. Duncan E. L. Ntzani E. E. (2012). Genome-wide meta-analysis identifies 56 bone mineral density loci and reveals 14 loci associated with risk of fracture. Nat. Genet. 44 (5), 491–501. 10.1038/ng.2249 22504420 PMC3338864

[B20] Fan B. Shepherd J. A. Levine M. A. Steinberg D. Wacker W. Barden H. S. (2014). National health and nutrition examination survey whole-body dual-energy X-Ray absorptiometry reference data for GE lunar systems. J. Clin. Densitom. 17(3), 344–377. 10.1016/j.jocd.2013.08.019 24161789

[B21] Fernandez-Rebollo E. Eipel M. Seefried L. Hoffmann P. Strathmann K. Jakob F. (2018). Primary osteoporosis is not reflected by disease-specific DNA methylation or accelerated epigenetic age in blood. J. Bone Miner Res. 33(2), 356–361. 10.1002/jbmr.3298 28926142

[B22] Gaunt T. R. Shihab H. A. Hemani G. Min J. L. Woodward G. Lyttleton O. (2016). Systematic identification of genetic influences on methylation across the human life course. Genome Biol. 17, 61. 10.1186/s13059-016-0926-z PMC4818469 27036880

[B23] Grundberg E. Meduri E. Sandling J. K. Hedman ÅK Keildson S. Buil A. (2013). Global analysis of DNA methylation variation in adipose tissue from twins reveals links to disease-associated variants in distal regulatory elements. Am. J. Hum. Genet. 93 (5), 876–890. 10.1016/j.ajhg.2013.10.004 24183450 PMC3824131

[B24] Hall D. B. (2000). Zero-inflated poisson and binomial regression with random effects: a case study. Biometrics. 56 (4), 1030–1039. 10.1111/j.0006-341X.2000.01030.x 11129458

[B25] He H. Liu Y. Tian Q. Papasian C. J. Hu T. Deng H. W. (2016). Relationship of sarcopenia and body composition with osteoporosis. Osteoporos Int. 27 (2), 473–482. 10.1007/s00198-015-3241-8 26243357

[B26] Hemani G. Zheng J. Elsworth B. Wade K. H. Haberland V. Baird D. (2018). The MR-Base platform supports systematic causal inference across the human phenome. Elife. 7, e34408. 10.7554/eLife.34408 29846171 PMC5976434

[B27] Hilbe J. M. (2009). Logistic regression models. (Boca Raton, Forida, USA: Chapman and hall/CRC). 10.1201/9781420075779

[B28] Houseman E. A. Accomando W. P. Koestler D. C. Christensen B. C. Marsit C. J. Nelson H. H. (2012). DNA methylation arrays as surrogate measures of cell mixture distribution. BMC Bioinf. 13, 86. 10.1186/1471-2105-13-86 PMC3532182 22568884

[B29] Kanis J. A. Melton L. J. Christiansen C. Johnston C. C. Khaltaev N. (1994). The diagnosis of osteoporosis. J. Bone Miner Res. 9 (8), 1137–1141. 10.1002/jbmr.5650090802 7976495

[B30] Kassahn K. S. Holmes O. Nones K. Patch A. M. Miller D. K. Christ A. N. (2013). Somatic point mutation calling in low cellularity tumors. PloS One. 8 (11), e74380. 10.1371/journal.pone.0074380 24250782 PMC3826759

[B31] Kelly T. L. Wilson K. E. Heymsfield S. B. (2009). Dual energy X-ray absorptiometry body composition reference values from NHANES. PloS One. 4(9), e7038. 10.1371/journal.pone.0007038 19753111 PMC2737140

[B32] Kemp J. P. Morris J. A. Medina-Gomez C. Forgetta V. Warrington N. M. Youlten S. E. (2017). Identification of 153 new loci associated with heel bone mineral density and functional involvement of GPC6 in osteoporosis. Nat. Genet. 49 (10), 1468–1475. 10.1038/ng.3949 28869591 PMC5621629

[B33] Kichaev G. Yang W. Y. Lindstrom S. Hormozdiari F. Eskin E. Price A. L. (2014). Integrating functional data to prioritize causal variants in statistical fine-mapping studies. PloS Genet. 10 (10), e1004722. 10.1371/journal.pgen.1004722 25357204 PMC4214605

[B34] Kichaev G. Roytman M. Johnson R. Eskin E. Lindström S. Kraft P. (2017). Improved methods for multi-trait fine mapping of pleiotropic risk loci. Bioinformatics. 33 (2), 248–255. 10.1093/bioinformatics/btw615 27663501 PMC5254076

[B35] Kim J. Wang Z. Heymsfield S. B. Baumgartner R. N. Gallagher D. (2002). Total-body skeletal muscle mass: estimation by a new dual-energy X-ray absorptiometry method. Am. J. Clin. Nutr. 76 (2), 378–383. 10.1093/ajcn/76.2.378 12145010

[B36] Kim S. Chen Z. Chamberlain N. D. Essani A. B. Volin M. V. Amin M. A. (2014). Ligation of TLR5 promotes myeloid cell infiltration and differentiation into mature osteoclasts in rheumatoid arthritis and experimental arthritis. J. Immunol. 193 (8), 3902–3913. 10.4049/jimmunol.1302998 25200955 PMC4185216

[B37] Klein H. U. Hebestreit K. (2016). An evaluation of methods to test predefined genomic regions for differential methylation in bisulfite sequencing data. Brief Bioinform. 17 (5), 796–807. 10.1093/bib/bbv095 26515532

[B38] Krueger F. Andrews S. R. (2011). Bismark: a flexible aligner and methylation caller for Bisulfite-Seq applications. Bioinformatics. 27 (11), 1571–1572. 10.1093/bioinformatics/btr167 PMC3102221 21493656

[B39] Kwok S. K. Cho M. L. Park M. K. Oh H. J. Park J. S. Her Y. M. (2012). Interleukin-21 promotes osteoclastogenesis in humans with rheumatoid arthritis and in mice with collagen-induced arthritis. Arthritis Rheumatol. 64(3), 740–750. 10.1002/art.33390 21968544

[B40] Li H. Durbin R. (2010). Fast and accurate long-read alignment with Burrows-Wheeler transform. Bioinformatics. 26 (5), 589–595. 10.1093/bioinformatics/btp698 20080505 PMC2828108

[B41] Li H. Handsaker B. Wysoker A. Fennell T. Ruan J. Homer N. (2009). The sequence alignment/map format and SAMtools. 25 (16), 2078–2079. Bioinformatics. 10.1093/bioinformatics/btp352 19505943 PMC2723002

[B42] Liu Y. Z. Dvornyk V. Lu Y. Shen H. Lappe J. M. Recker R. R. (2005). A novel pathophysiological mechanism for osteoporosis suggested by an *in vivo* gene expression study of circulating monocytes. J. Biol. Chem. 280 (32), 29011–29016. 10.1074/jbc.M501164200 15965235

[B43] Liu Y. Aryee M. J. Padyukov L. Fallin M. D. Hesselberg E. Runarsson A. (2013). Epigenome-wide association data implicate DNA methylation as an intermediary of genetic risk in rheumatoid arthritis. Nat. Biotechnol. 31 (2), 142–147. 10.1038/nbt.2487 23334450 PMC3598632

[B44] Lizio M. Harshbarger J. Abugessaisa I. Noguchi S. Kondo A. Severin J. (2017). Update of the FANTOM web resource: high resolution transcriptome of diverse cell types in mammals. Nucleic Acids Res. 45 (D1), D737–D743. 10.1093/nar/gkw995 27794045 PMC5210666

[B45] MacArthur J. Bowler E. Cerezo M. Gil L. Hall P. Hastings E. (2017). The new NHGRI-EBI Catalog of published genome-wide association studies (GWAS Catalog). Nucleic Acids Res. 45 (D1), D896–D901. 10.1093/nar/gkw1133 27899670 PMC5210590

[B46] Marini F. Cianferotti L. Brandi M. L. (2016). Epigenetic mechanisms in bone biology and osteoporosis: can they drive therapeutic choices? Int. J. Mol. Sci. 17 (8), E1329. 10.3390/ijms17081329 27529237 PMC5000726

[B47] Martin M. (2011). Cutadapt removes adapter sequences from high-throughput sequencing reads. EMBnet. J. 17 (1), 10. 10.14806/ej.17.1.200

[B48] McClay J. L. Shabalin A. A. Dozmorov M. G. Adkins D. E. Kumar G. Nerella S. (2015). High density methylation QTL analysis in human blood *via* next-generation sequencing of the methylated genomic DNA fraction. Genome Biol. 16, 291. 10.1186/s13059-015-0842-7 PMC4699364 26699738

[B49] McCullagh P. (2019). Generalized linear models. (Abingdon, UK: Routledge). 10.1201/9780203753736

[B50] McKenna A. Hanna M. Banks E. Sivachenko A. Cibulskis K. Kernytsky A. (2010). The genome analysis toolkit: a MapReduce framework for analyzing next-generation DNA sequencing data. Genome Res. 20 (9), 1297–1303. 10.1101/gr.107524.110 20644199 PMC2928508

[B51] McLaren W. Gil L. Hunt S. E. Riat H. S. Ritchie G. R. S. Thormann A. (2016). The ensembl variant effect predictor. Genome Biol. 17, 122. 10.1186/s13059-016-0974-4 PMC4893825 27268795

[B52] Meissner A. Gnirke A. Bell G. W. Ramsahoye B. Lander E. S. Jaenisch R. (2005). Reduced representation bisulfite sequencing for comparative high-resolution DNA methylation analysis. Nucleic Acids Res. 33 (18), 5868–5877. 10.1093/nar/gki901 16224102 PMC1258174

[B53] Morris J. A. Tsai P. C. Joehanes R. Zheng J. Trajanoska K. Soerensen M. (2017). Epigenome-wide association of DNA methylation in whole blood with bone mineral density. J. Bone Miner Res. 32 (8), 1644–1650. 10.1002/jbmr.3148 28394087 PMC5615229

[B54] Morris J. A. Kemp J. P. Youlten S. E. Laurent L. Logan J. G. Chai R. C. (2019). An atlas of genetic influences on osteoporosis in humans and mice. Nat. Genet. 51 (2), 258–266. 10.1038/s41588-018-0302-x 30598549 PMC6358485

[B55] Nagy Z. B. Gergely P. Donáth J. Borgulya G. Csanád M. Poór G. (2008). Gene expression profiling in Paget's disease of bone: upregulation of interferon signaling pathways in pagetic monocytes and lymphocytes. J. Bone Miner Res. 23 (2), 253–259. 10.1359/jbmr.071021 18197754

[B56] Nose M. Yamazaki H. Hagino H. Morio Y. Hayashi S. I. Teshima R. (2009). Comparison of osteoclast precursors in peripheral blood mononuclear cells from rheumatoid arthritis and osteoporosis patients. J. Bone Miner Metab. 27 (1), 57–65. 10.1007/s00774-008-0011-0 19082778

[B57] Palmer T. M. Lawlor D. A. Harbord R. M. Sheehan N. A. Tobias J. H. Timpson N. J. (2012). “Using multiple genetic variants as instrumental variables for modifiable risk factors,” Statistical Methods in Medical Research. 21 (3), 223–242. 10.1177/0962280210394459 21216802 PMC3917707

[B58] Parhami F. Garfinkel A. Demer L. L. (2000). Role of lipids in osteoporosis. Arteriosclerosis Thrombosis Vasc. Biol. 20 (11), 2346–2348. 10.1161/01.ATV.20.11.2346 11073836

[B59] Pierce B. L. Ahsan H. Vanderweele T. J. (2011). Power and instrument strength requirements for Mendelian randomization studies using multiple genetic variants. Int. J. Epidemiol. 40 (3), 740–752. 10.1093/ije/dyq151 20813862 PMC3147064

[B60] Price E. M. Robinson W. P. (2018). Adjusting for batch effects in DNA methylation microarray data, a lesson learned. Front. Genet. 16 (9), 83. 10.3389/fgene.2018.00083 PMC5864890 29616078

[B61] Price A. L. Patterson N. J. Plenge R. M. Weinblatt M. E. Shadick N. A. Reich D. (2006). Principal components analysis corrects for stratification in genome-wide association studies. Nat. Genet. 38 (8), 904–909 10.1038/ng1847 16862161

[B62] Ralston S. H. Uitterlinden A. G. (2010). Genetics of osteoporosis. Endocr. Rev. 31 (5), 629–662. 10.1210/er.2009-0044 20431112

[B63] Relton C. L. Davey Smith G. (2012). Two-step epigenetic mendelian randomization: a strategy for establishing the causal role of epigenetic processes in pathways to disease. Int. J. Epidemiol. 41 (1), 161–176. 10.1093/ije/dyr233 22422451 PMC3304531

[B64] Reppe S. Lien T. G. Hsu Y. H. Gautvik V. T. Olstad O. K. Yu R. (2017). Distinct DNA methylation profiles in bone and blood of osteoporotic and healthy postmenopausal women. Epigenetics. 12 (8), 647–687. 10.1080/15592294.2017.1345832 PMC5687328 28650214

[B65] Richardson T. G. Zheng J. Davey Smith G. Timpson N. J. Gaunt T. R. Relton C. L. (2017). Mendelian randomization analysis identifies CpG sites as putative mediators for genetic influences on cardiovascular disease risk. Am. J. Hum. Genet. 101 (4), 590–602. 10.1016/j.ajhg.2017.09.003 28985495 PMC5630190

[B66] Rivadeneira F. Styrkársdottir U. Estrada K. Halldórsson B. V. Hsu Y. H. Richards J. B. (2009). Twenty bone-mineral-density loci identified by large-scale meta-analysis of genome-wide association studies. Nat. Genet. 41(11), 1199–1206. 10.1038/ng.446 19801982 PMC2783489

[B67] Roadmap Epigenomics Consortium Kundaje A. Meuleman W. Ernst J. Bilenky M. Yen A. (2015). Integrative analysis of 111 reference human epigenomes. Nature 518 (7539), 317–29. 10.1038/nature14248 PMC4530010 25693563

[B68] Sabik O. L. Farber C. R. (2017). Using GWAS to identify novel therapeutic targets for osteoporosis. Trans. Res. 181, 15–26. 10.1016/j.trsl.2016.10.009 PMC5357198 27837649

[B69] Schartner C. Ziegler C. Schiele M. A. Kollert L. Weber H. Zwanzger P. (2017). CRHR1 promoter hypomethylation: an epigenetic readout of panic disorder? Eur. Neuropsychopharmacol. 27 (4), 360–371. 10.1016/j.euroneuro.2017.01.005 28233670

[B70] Shahi M. Peymani A. Sahmani M. (2017). Regulation of bone metabolism. Rep. Biochem. Mol. Biol. 5 (2), 73–82. 28367467 PMC5346273

[B71] Styrkarsdottir U. Halldorsson B. V. Gudbjartsson D. F. Tang N. L. S. Koh J. M. Xiao S. M. (2010). European bone mineral density loci are also associated with BMD in East-Asian populations. PloS One. 5 (10), e13217. 10.1371/journal.pone.0013217 PMC2951352 20949110

[B72] Sun Y. V. Turner S. T. Smith J. A. Hammond P. I. Lazarus A. Van De Rostyne J. L. (2010). Comparison of the DNA methylation profiles of human peripheral blood cells and transformed B-lymphocytes. Hum. Genet. 127 (6), 651–658. 10.1007/s00439-010-0810-y 20238126 PMC2873107

[B73] Sweeney J. Haslett J. Parnell A. C. (2014). The zero & $ N $-inflated binomial distribution with applications. arXiv Prepr arXiv14070064.

[B74] van Meurs J. B. J. Boer C. G. Lopez-Delgado L. Riancho J. A. (2019). Role of epigenomics in bone and cartilage disease. J. Bone Miner Res. 34 (2), 215–230. 10.1002/jbmr.3662 30715766

[B75] Verbanck M. Chen C. Y. Neale B. Do R. (2018). Detection of widespread horizontal pleiotropy in causal relationships inferred from Mendelian randomization between complex traits and diseases. Nat. Genet. 50 (5), 693–698. 10.1038/s41588-018-0099-7 29686387 PMC6083837

[B76] Vimaleswaran K. S. Berry D. J. Lu C. Tikkanen E. Pilz S. Hiraki L. T. (2013). Causal relationship between obesity and vitamin D status: bi-directional mendelian randomization analysis of multiple cohorts. PloS Med. 10 (2), e1001383 10.1371/journal.pmed.1001383 PMC3564800 23393431

[B77] Wahl S. Drong A. Lehne B. Loh M. Scott W. R. Kunze S. (2017). Epigenome-wide association study of body mass index, and the adverse outcomes of adiposity. Nature. 541 (7635), 81–86. 10.1038/nature20784 28002404 PMC5570525

[B78] Wong K. L. Tai J. J. Y. Wong W. C. Han H. Sem X. Yeap W. H. (2011). Gene expression profiling reveals the defining features of the classical, intermediate,and nonclassical human monocyte subsets. Blood. 118(5), e16–e31. 10.1182/blood-2010-12-326355 21653326

[B79] Wright F. A. Sullivan P. F. Brooks A. I. Zou F. Sun W. Xia K. (2014). Heritability and genomics of gene expression in peripheral blood. Nat. Genet. 46 (5), 430–437. 10.1038/ng.2951 24728292 PMC4012342

[B80] Xing L. Schwarz E. M. Boyce B. F. (2005). Osteoclast precursors, RANKL/RANK, and immunology. Immunol. Rev. 208 (1), 19–29. 10.1111/j.0105-2896.2005.00336.x 16313338

[B81] Zheng H. Forgetta V. Hsu Y. Estrada K. Rosello-Diez A. Leo P. J. (2015). Whole-genome sequencing identifies EN1 as a determinant of bone density and fracture. Nature 526 (7571), 112–117. 10.1038/nature14878 26367794 PMC4755714

[B82] Zhou Y. Deng H. W. Shen H. (2015). Circulating monocytes: an appropriate model for bone-related study. Osteoporosis Int. 26 (11), 2561–2572 10.1007/s00198-015-3250-7 26194495

[B83] Zhou Y. Zhou B. Pache L. Chang M. Khodabakhshi A. H. Tanaseichuk O. (2019). Metascape provides a biologist-oriented resource for the analysis of systems-level datasets. Nat. Commun. 10 (1), 1523. 10.1038/s41467-019-09234-6 PMC6447622 30944313

[B84] Zhou X. Qiu Y. H. He P. Jiang F. Wu L. F. Lu X. (2019). Why SNP rs227584 is associated with human BMD and fracture risk? A molecular and cellular study in bone cells. J. Cell Mol. Med. 23 (2), 898–907. 10.1111/jcmm.13991 30370607 PMC6349212

[B85] Zhu Z. Zhang F. Hu H. Bakshi A. Robinson M. R. Powell J. E. (2016). Integration of summary data from GWAS and eQTL studies predicts complex trait gene targets. Nat. Genet. 48 (5), 481–487. 10.1038/ng.3538 27019110

[B86] Ziller M. J. Hansen K. D. Meissner A. Aryee M. J. (2015). Coverage recommendations for methylation analysis by whole-genome bisulfite sequencing. Nat. Methods. 12 (3), 230–232. 10.1038/nmeth.3152 25362363 PMC4344394

