# Supplementary material for: Mendelian Randomization Identifies CpG Methylation Sites With Mediation Effects for Genetic Influences on BMD in Peripheral Blood Monocytes
Source: Front Genet. 2020 Feb 28;11:60. doi: 10.3389/fgene.2020.00060 (PMC7059767; doi:10.3389/fgene.2020.00060)
Supplement: Supplementary file 2 [file Presentation_1.pdf]

Supplementary Materials for the paper

Mendelian Randomization Identifies CpG Methylation Sites with Mediation Effects for Genetic Influences on BMD in Peripheral Blood Monocytes

Part 1. Power analysis of using logistic regression to detect DMC

We performed simulation analysis to calculate the power of detecting methylation difference great than 0.05 using logistic regression. We considered the following two different scenarios:

In scenario 1, the ground truth is that the difference in methylation statistically caused solely by BMD group is 0.05. To make it concise, we use a model of simple logistic regression, in which the only predictor variable of DNA methylation value at a CpG site is the BMD group. However, this scenario could also represent the case that one or more covariates have been adjusted.

In scenario 2, the total effect of BMD and other covariates on the mean difference in methylation is set to be 0.05. We used a model of multiple logistic regression, which contains five predictor variables (age, BMI, drinking status, smoking status, and 1st PC of methylation), same as what we used in the real differential methylation analysis.

At a CpG locus, the methylation data y_{ij} of subject i group j is simulated by equation (1)(2), where z_{ijn} is the methylation status of subject i group j sequence n; $i = 1,2 \dots I_j; I_0 = 54; I_1 = 64;$ $j = 0,1;$ $n = 1, \dots, N_{ij}$. In both scenarios, we control two parameters that affect the power, the coverage of the CpG site in each subject (N_{ij}) and the probability of methylation in the low BMD group (p_0). The group difference is set to be 0.05, i.e. $p_1 = p_0 + 0.05$.

$$y_{ij} = \sum_{n=1}^{N_i} z_{ijn} \sim binomial\left(N_i, p_j\right) \quad (1)$$

$$z_{ijn} \sim Bernoulli\left(p_j\right) \qquad (2)$$

The logistic regression in equation (3) was then fitted for the simulated data. The covariate part was omitted in scenario 1. In scenario 2, five covariates are simulated according to the group-specific parameters in **Table 1**. For each parameter setting, the regression was replicated 1000 times. The empirical power is calculated as the number of times the null hypothesis (H_0: $\delta = 0$) is rejected over the number of replications. It is important to keep in mind that the sample size of

(3) is the number of z_{ijn} instead of y_{ij}, thus the sample size is $\sum_{ij} N_{ij}$. This fact has also been pointed out in the original paper of *methylKit* package.

$$\log\frac{p}{1-p} = \beta_0 + \delta BMD \quad + \sum\nolimits_{m=1}^{5} \beta_m x_m \qquad (3)$$

Figure S5-a shows the power versus p_0 (varies from 0.1 to 0.9) at a mean coverage of 30 in the two scenarios. In scenario 1, the power of detecting 0.05 difference caused by BMD group is constantly greater than 80% regardless of the change of p_0. The power curve makes a U-shape with lower power at p_0=0.5 and higher power at p_0=0.1 and 0.9. In scenario 2, the power is very close to 80% for detecting the methylation difference in the BMD group adjusting for other covariates. There's a U-shape in the power curve but not as apparent as the simple logistic regression model (scenario 1).

Figure S5-b shows the power versus mean coverage with p_0=0.5 in the two scenarios. The power increases in both scenarios with the increase of mean coverage. The power curve vs coverage at p_0 of other values should be superior to the power curve shown in Figure S5-b, because power at p_0=0.5 is the lowest as we demonstrated in Figure 5-a.

Part 2. Supplementary figures

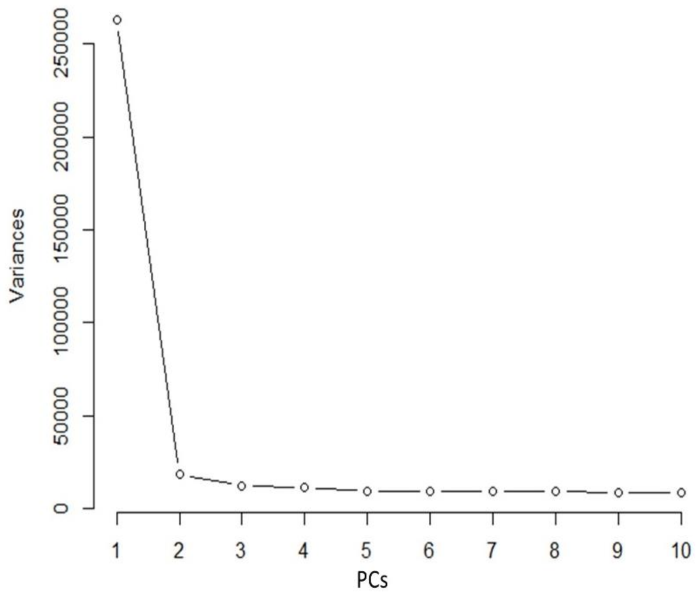

Figure S1. Scree Plot of Principal Component of Methylation

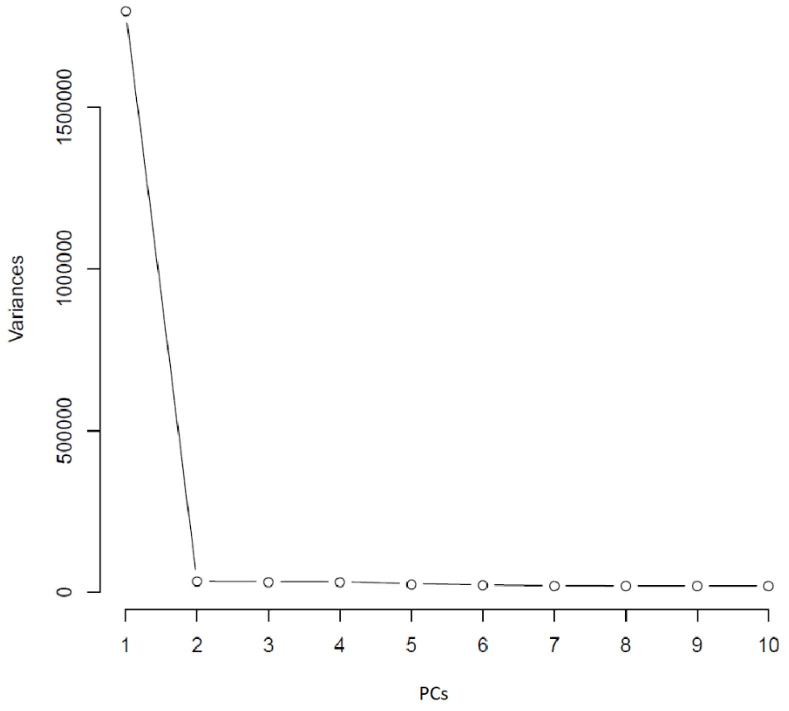

Figure S2. Scree Plot of the Principal of Components of SNP

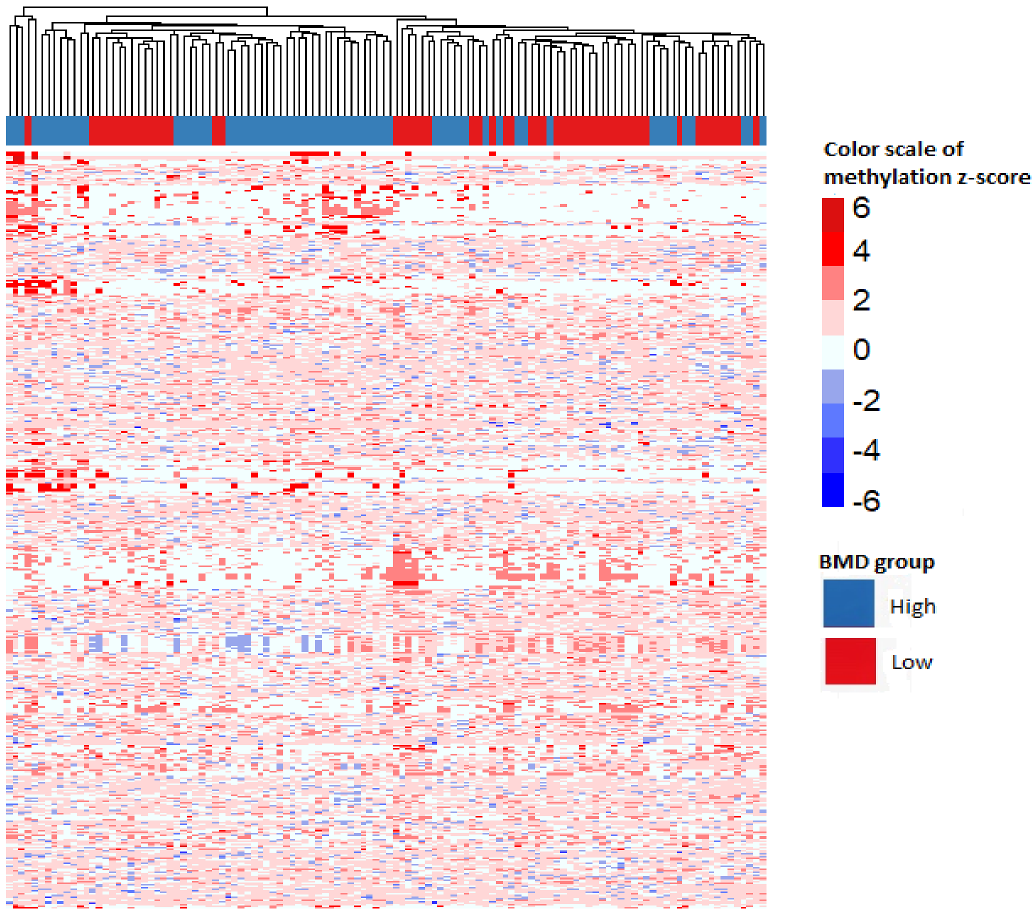

Figure S3. Heatmap of the methylation values of the 693 DMC with mean difference ≥ 0.1. The rows represent CpG loci and the columns represent subjects. The methylation level in each row is scaled to a Z-score. Both row and column are clustered by 'hclust' algorithm by R package *pheatmap*.

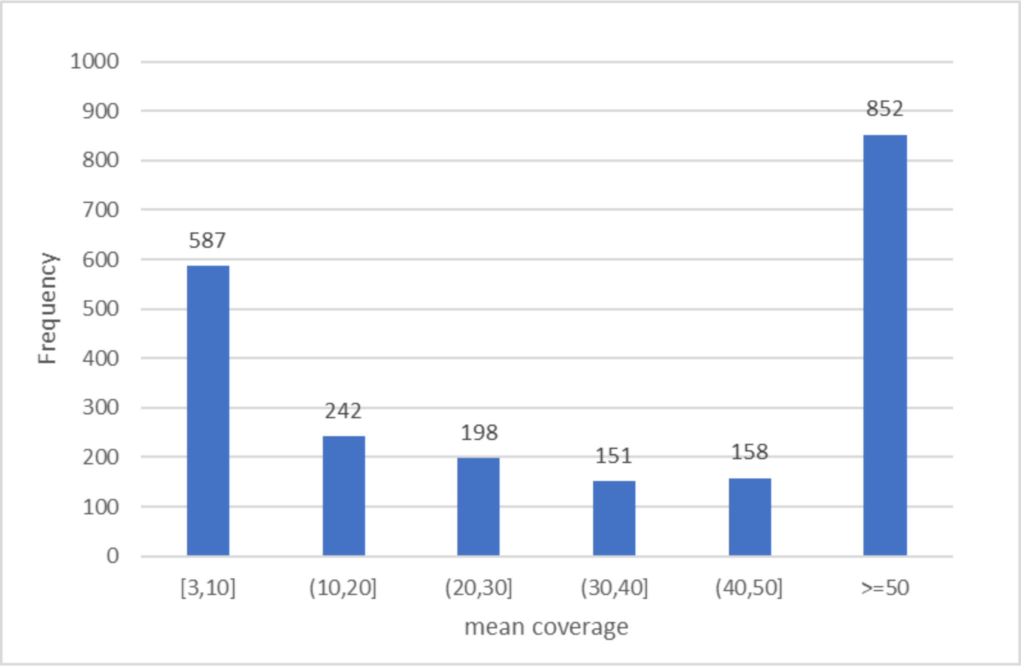

Figure S4. Histogram of mean coverage of DMCs with difference > 0.05.

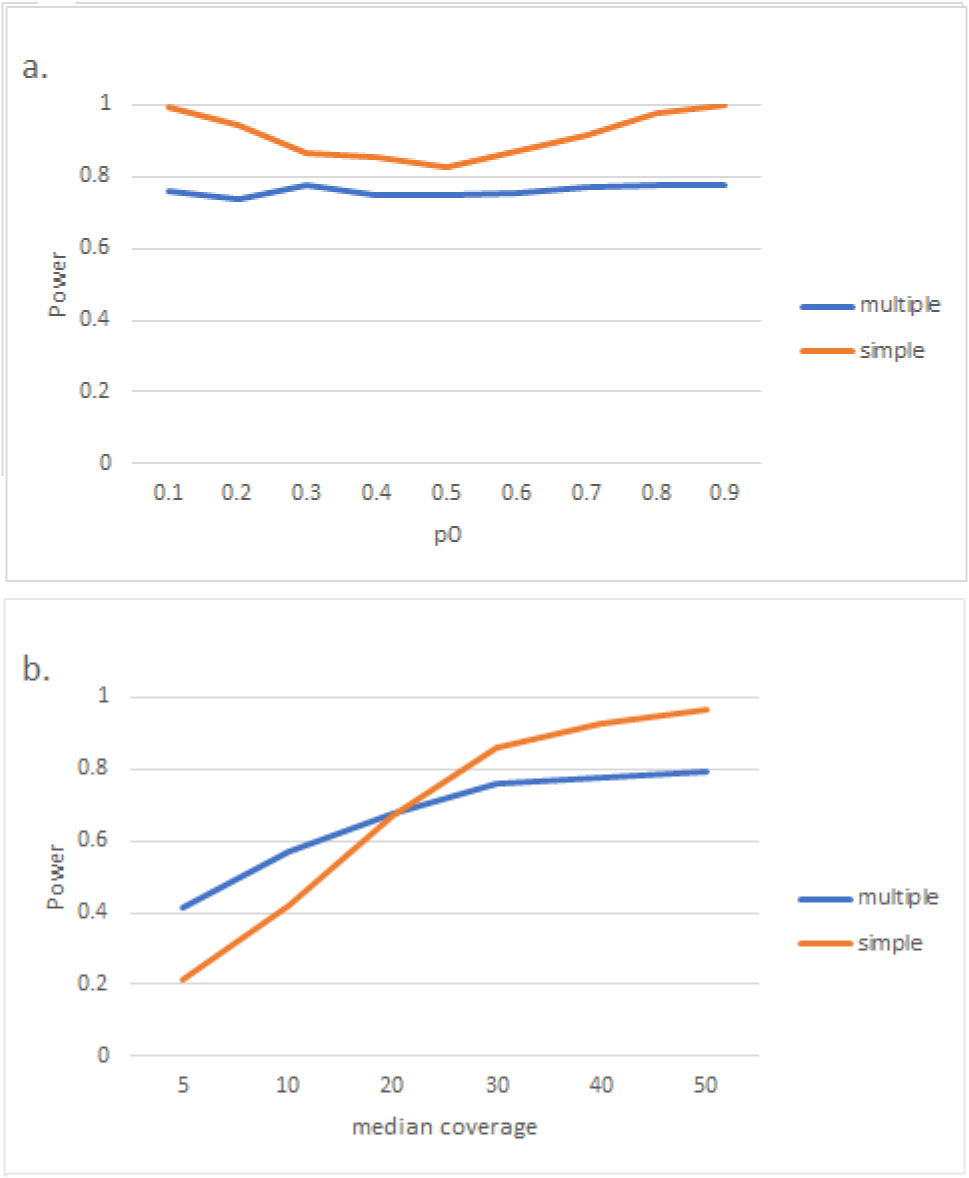

Figure S5. Plot of power versus a. p_0 at a mean coverage of 30; b. mean coverage at p_0 = 0.5 in the two scenarios.

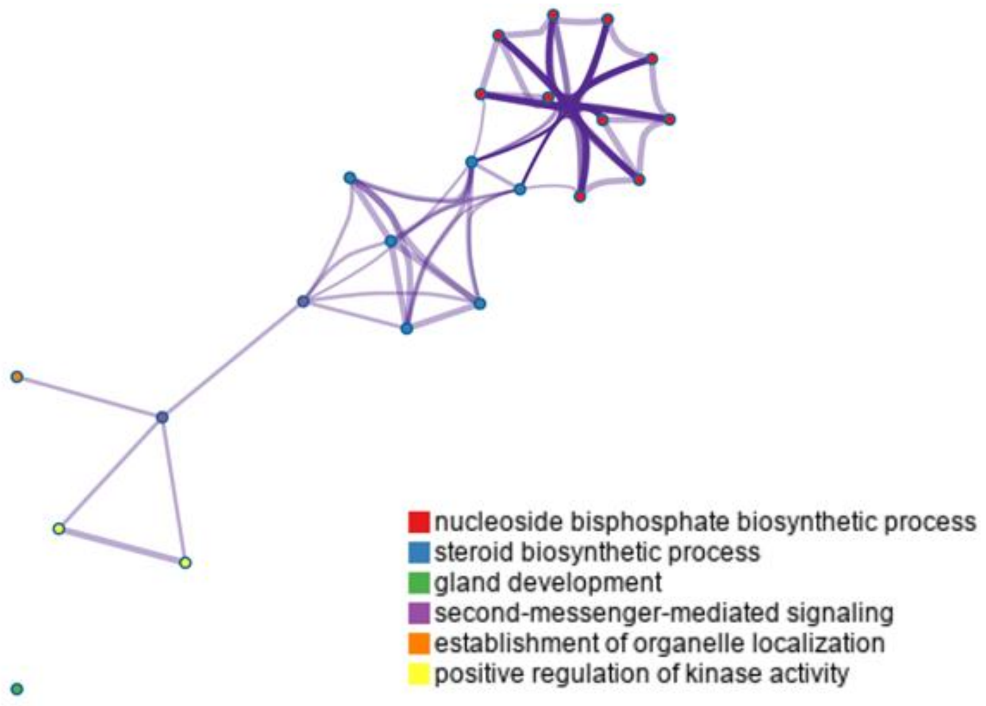

Figure S6. Network of enriched terms. Each node represents an enriched term and is colored by its cluster name.

Part 3. Supplementary tables

Table S13. Top six clusters with their representative enriched GO terms. "Count" is the number of genes in the user-provided lists with membership in the given ontology term. "%" is the percentage of all of the user-provided genes that are found in the given ontology.

GO	Category	Description	Count	%	Log10(P)	Log10(q)
**GO:0033866**	GO Biological Processes	nucleoside bisphosphate biosynthetic process	3	17.65	-4.85	-1.01
**GO:0006694**	GO Biological Processes	steroid biosynthetic process	3	17.65	-3.48	-0.07
**GO:0048732**	GO Biological Processes	gland development	3	17.65	-2.49	0.00
**GO:0019932**	GO Biological Processes	second-messenger-mediated signaling	3	17.65	-2.47	0.00
**GO:0051656**	GO Biological Processes	establishment of organelle localization	3	17.65	-2.29	0.00
**GO:0033674**	GO Biological Processes	positive regulation of kinase activity	3	17.65	-2.15	0.00

Table S14. Metrics of overdispersion, zero-inflation and mean coverage of the 30 identified mediator DMCs

chr	start	prob	varEs	varOb	scalar	coverage	nZero	Gene
**1**	22571385	0.62	27.66	350.12	12.66	35.46	12	MIR4418
**2**	31719473	0.81	21.21	698.75	32.95	78.73	2	SRD5A2
**4**	957285	0.78	11.02	150.67	13.67	24.29	5	DGKQ/SLC26A1
**6**	32222711	0.79	160.94	22254.76	138.28	307.54	0	XXbac-BPG154L12.4
**7**	1062527	0.44	59.75	2112.08	35.35	105.18	31	C7orf50
**7**	56435427	0.59	103.36	1919.44	18.57	122.92	9	RP11-700P18.1
**8**	58127658	0.83	87.69	17359.24	197.97	372.78	0	RP11-513O17.2
**9**	71682281	0.57	50.40	1649.50	32.73	91.58	10	PRKACG
**9**	96362114	0.63	54.39	2147.44	39.48	114.21	9	PHF2
**10**	42739065	0.21	147.05	3509.43	23.87	301.19	0	RP11-313J2.1
**12**	48723325	0.72	129.87	25037.68	192.79	283.64	7	SENP1
**14**	75441795	0.44	37.03	707.09	19.09	60.42	23	TMED10
**16**	81248716	0.59	124.57	7322.34	58.78	251.15	3	PKD1L2
**16**	89167395	0.18	11.88	102.84	8.66	36.37	68	ACSF3
**17**	1944903	0.06	22.58	487.86	21.61	181.55	46	DPH1
**17**	1944905	0.06	24.00	486.42	20.26	181.45	40	DPH1
**17**	42246289	0.57	11.28	55.42	4.91	19.91	8	C17orf53
**17**	43828617	0.81	18.98	522.06	27.51	48.86	4	MAPT
**17**	43894548	0.78	5.57	20.26	3.64	8.98	6	CRHR1
**17**	44060776	0.80	5.36	41.00	7.65	12.76	5	ARHGAP27
**17**	44337590	0.07	47.58	1040.95	21.88	238.20	61	ARL17B
**17**	44337597	0.07	47.84	1017.07	21.26	238.38	60	ARL17B
**17**	44337604	0.07	47.59	1005.52	21.13	238.07	57	ARL17B
**17**	44337613	0.07	47.82	1021.04	21.35	238.03	61	ARL17B
**17**	44337617	0.08	49.55	1035.92	20.91	238.09	46	RP11-995C19.2
**17**	44337622	0.08	48.32	1016.35	21.04	237.69	45	RP11-995C19.2
**17**	80053590	0.26	28.69	43.75	1.52	23.38	35	FASN
**17**	80086159	0.49	28.98	651.03	22.46	54.62	27	FASN
**21**	46677414	0.52	32.45	245.25	7.56	46.62	1	LINC00334
**22**	46504167	0.40	94.62	492.30	5.20	102.52	5	FLJ27365

Columns
1 chromosome, 2 base position, 3 mean methylation level, 4 estimated variance, 5 observed variance, 6 overdispersion scalar, 7 mean coverage, 8 number of zeros, 9 if the evidence of association is low, 10 causal mQTL overlapped gene
